# Modulation of the *Pseudomonas aeruginosa* quorum sensing cascade by MexT-regulated factors

**DOI:** 10.1128/mbio.02941-25

**Published:** 2025-10-23

**Authors:** Andrew Frando, Robert S. Parsek, Jamal Omar, Marie-Christine Groleau, Mylène C. Trottier, Nicole E. Smalley, Eric Déziel, Ajai A. Dandekar

**Affiliations:** 1 Department of Medicine, University of Washington 7284, Seattle, Washington, USA; 2 Department of Microbiology, University of Washington 7284, Seattle, Washington, USA; 3 Centre Armand-Frappier Santé Biotechnologie, Institut National de la Recherche Scientifique (INRS) 14851, Laval, Québec, Canada; Georgia Institute of Technology, Atlanta, Georgia, USA

**Keywords:** LasR, RhlR, PqsR, efflux

## Abstract

*Pseudomonas aeruginosa (Pa*) uses quorum sensing (QS), a cell-cell communication system that enables it to sense cell density and alter gene expression. *Pa* has three complete QS circuits controlled by the regulators LasR, RhlR, and PqsR, which together activate the transcription of hundreds of genes. In the well-studied strain PAO1, QS is organized hierarchically, with PqsR and RhlR activity dependent on LasR. This hierarchy depends on the transcription factor MexT; deletion of *mexT* allows for RhlR activity in the absence of LasR. We aimed to identify how MexT modulates *Pa* QS architecture. We compared the transcriptome of PAO1 to that of PAO1Δ*mexT* and determined a MexT regulon. We identified two MexT-regulated operons that may affect the QS hierarchy: the efflux pump genes *mexEF-oprN* and the *Pseudomonas* quinolone signal (PQS) synthesis genes *pqsABCDE*. We tested whether the products of these genes affected the QS hierarchy. A *mexEF* knockout mutant, like a *mexT* deletion mutant, exhibited RhlR activity earlier and to a higher magnitude than wild-type PAO1. MexEF-OprN is known to export quinolones, and we found that exogenous addition of PQS also resulted in earlier and higher magnitude of RhlR activity, through PqsE, compared with wild-type PAO1. We also discovered alternate QS architectures in clinical isolates, where RhlR activity is not fully dependent on LasR. In these isolates, MexT does not influence the relationship between LasR and RhlR. Our work reveals a new suite of factors that regulate QS in *Pa*, with implications for bacterial behaviors in environmental and clinical settings.

**IMPORTANCE:**

Bacteria interact with both abiotic and biotic factors in their environment. Quorum sensing (QS) is one mechanism that bacteria use to communicate with other bacteria and coordinate behaviors in the population. QS regulates a wide variety of processes ranging from the production of light to the modulation of virulence factors; some bacteria use single QS circuits, whereas others have several. The opportunistic pathogen *Pseudomonas aeruginosa* uses QS to control some virulence functions and has three complete QS circuits. Our study explores why bacteria might have multiple QS circuits. We show how a non-QS regulated factor, MexT, influences QS regulators in *P. aeruginosa,* and we uncover the diversity of QS architectures in clinical isolates. These studies begin to reveal the benefits (or disadvantages) of multiple QS circuits, allowing us to understand the behaviors of bacteria that have a range of implications in health, agriculture, and bioremediation.

## INTRODUCTION

Bacteria can occupy a variety of environments that include the ocean, human body, and soil, and seldom live in isolation. Bacteria have many methods of adapting to environmental changes and interacting with other microbes by using various forms of signaling. Quorum sensing (QS) is a type of intercellular communication used by many bacteria to coordinate group behaviors in response to cell density. QS is a feature of many Gram-positive and Gram-negative bacteria and typically results in transcription factor activation of gene expression (1). Bacterial behaviors regulated by QS include the production of light, biofilm formation, and the secretion of virulence factors.

QS is typically organized into circuits composed of a signal-producing enzyme and a signal-responsive transcriptional regulator. *Pseudomonas aeruginosa* (*Pa*) is a Gram-negative opportunistic bacterial pathogen encoding multiple QS circuits that are controlled by the transcriptional regulators LasR, RhlR, and PqsR (also called MvfR) (2). Together, the three QS circuits regulate 5% of *P. aeruginosa* genes, including virulence factors like proteases, toxins, and biofilm components (3).

LasR and RhlR are both QS transcription factors that respond to acyl-homoserine lactone (AHL) signals (4). LasI produces the signal *N*-3-oxo-dodecanoyl homoserine lactone (3OC12-HSL) that binds to, and affects, LasR activity. Similarly, RhlI produces the signal *N-*butanoyl homoserine lactone (C4-HSL) that binds to RhlR. The PqsR QS circuit involves the alkyl-quinolone signals 2-heptyl-4-quinolone (HHQ) and 2-heptyl-3-hydroxy-4(1*H*)-quinolone (PQS) (5–8). In a process different from AHL QS, HHQ and PQS are synthesized from multiple enzymes encoded by *pqsABCD* and *pqsH*. All three circuits exhibit a positive feedback loop where the signal synthase enzymes produce signals that bind and activate the transcriptional regulator, which in turn results in an increased expression of the signal synthase enzymes (9), a typical feature of QS circuits.

The interconnectivity between the QS circuits, or QS architecture, differs between *Pa* strains. In the laboratory strain PAO1, QS architecture is hierarchical, where PqsR and RhlR expression are dependent on LasR (10). A *lasR*-null strain in PAO1 is generally in a QS-off state (11). There are additional layers of interaction: for instance, the chaperone-like protein PqsE, which is regulated by PQS QS, positively affects RhlR activity, whereas RhlR negatively regulates the production of PQS (12–14). The QS architecture in some clinical isolates varies from that of PAO1. We and others have found that despite encoding inactivating mutations in *lasR*, *Pa* clinical isolates are capable of activating RhlR QS independent of LasR, in a manner that differs from PAO1 (15–18). LasR-independent RhlR QS can also be found in other environmental contexts, including soil, hospital sink drains, and meat/fish markets (19). The question remains whether these isolates have QS architectures that are fundamentally different from PAO1 or if the phenotype is explained, instead, by mutations that were acquired in addition to those in *lasR*.

More recent work has identified how PAO1 can itself develop a LasR-independent QS architecture. The “wildtype” strain PAO1 acquired a loss-of-function mutation in the oxidoreductase gene *mexS*, presumably as a consequence of exposure to chloramphenicol (20–22). MexS responds to oxidative stress and negatively regulates the activity of a transcriptional regulator, MexT. Because of the inactivating mutation in *mexS,* MexT is constitutively active in PAO1 and induces many genes, including the RND drug efflux pump MexEF-OprN. Such *nfxC*-type multidrug-resistant strains that constitutively express *mexEF-oprN* due to mutations in *mexS* are commonly isolated, for instance, from airways of CF patients (22, 23). Recent work has linked MexT to the regulation of QS in PAO1 (24–26). MexEF-OprN influences QS, in part by delaying the activation of PQS QS (27). PAO1 with inactivating mutations in *lasR* does not activate RhlR QS. However, *lasR* mutants exhibit RhlR activity if there is also an inactivating mutation in the *mexT*. These data indicate that MexT contributes to the LasR-dependent QS architecture in PAO1, but the mechanisms driving this phenomenon are unknown.

We are interested in QS architecture and how these architectures are regulated, with the idea that the arrangement of QS circuits might impact bacterial virulence, social behaviors, or both. To investigate this issue, we explored how MexT regulates QS in the laboratory strain PAO1. We determined the MexT regulon by comparing the transcriptomes of wild-type PAO1 to a *mexT*-null mutant. We ascertained that the efflux pump MexEF-OprN and the chaperone protein PqsE both contributed to the LasR-dependent QS architecture in PAO1. To study the diversity of QS architectures, we next explored this aspect in clinical isolates with functional LasR alleles. We identified isolates with a PAO1-like QS architecture where RhlR activity is dependent, to varying degrees, on intact LasR, expanding the array of known QS architectures in *Pa*. Finally, we determined that MexT did not influence QS architecture in some of these clinical isolates, suggesting the existence of other regulators of the QS hierarchy in *Pa*.

## RESULTS

### The MexT regulon

Because inactivating mutations in *mexT* alter the QS hierarchy in PAO1, we started by exploring how MexT regulates QS architecture in this strain. MexT is a transcription factor that regulates several genes (28, 29), although the full extent of its gene regulation is not fully known, because a prior study compared the wild-type with an overexpression variant (29), and we now understand that MexT is constitutively expressed in PAO1. We aimed to identify MexT-regulated genes that modulate QS, specifically components of the MexT regulon that might impact the timing of RhlR QS activation.

**Fig 1:**
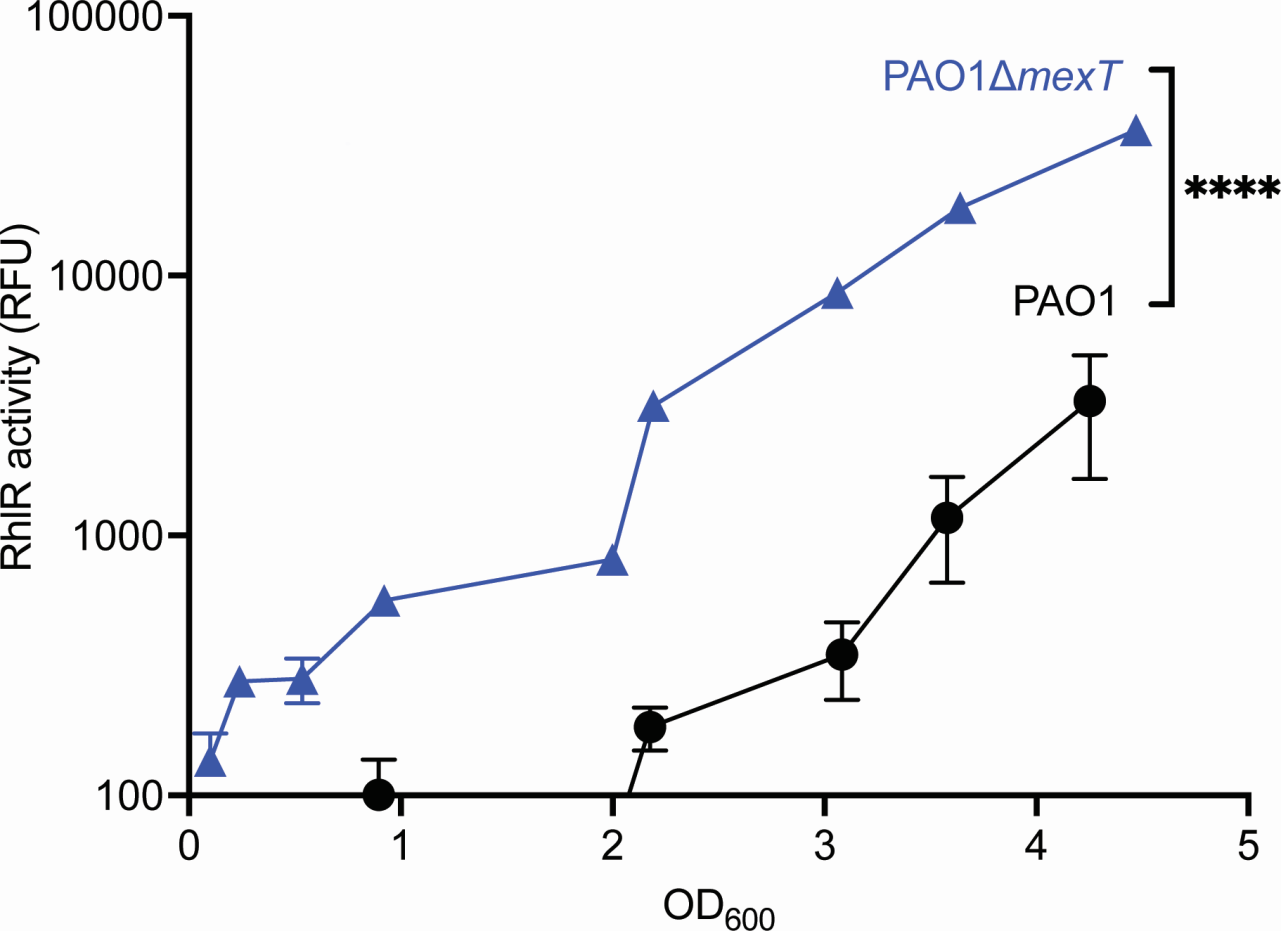
PAO1∆*mexT* exhibits earlier and greater RhlR activity than the wild-type. Wild-type PAO1 and PAO1∆*mexT* were grown in flasks for 4.5 h. Bacterial growth was determined by OD_600_, and RhlR activity was determined using a reporter plasmid with the promoter of the RhlR-regulated gene *rhlA* controlling GFP expression. The *x*-axis shows OD_600_, and the *y*-axis shows RhlR activity as measured using the *rhlA-gfp* reporter plasmid. The experiment was performed in triplicate. Error bars represent the standard deviation. *P* values were calculated using a two-way ANOVA with Geisser-Greenhouse correction, where all strains were compared with wild-type PAO1. **** denotes a *P* < 0.0001.

We compared RhlR activity in a PAO1 *mexT* knockout mutant (PAO1∆*mexT*), which does not express *mexT*, with that of wild-type PAO1, which expresses it constitutively (Fig. 1). In agreement with prior studies (24, 25), we found that the PAO1∆*mexT* mutant exhibited higher RhlR activity at lower cell densities compared with wild-type. The PAO1∆*mexT* knockout mutant has robust RhlR activity as early as an OD_600_ of 1.0, whereas wild-type PAO1 exhibits RhlR activity around an OD_600_ of 2.0. We identified the OD_600_ of 1.0 and 2.0 as the specific growth stages we would use to identify MexT-regulated genes that might modulate RhlR QS. We chose an OD_600_ of 1.0 as the PAO1∆*mexT* knockout mutant exhibited detectable RhlR activity at this cell density, whereas wild-type PAO1 did not (Fig. 1). We additionally chose the OD_600_ of 2.0 in case an OD_600_ of 1.0 was too early to capture some MexT-regulated genes, and this is the cell density at which many QS regulons have been defined (3, 30).

We performed an RNA-sequencing experiment using wild-type PAO1 and a *mexT* knockout mutant to identify differentially expressed genes (DEGs) at OD_600_ of 1.0 and 2.0. We compared gene expression between the two strains using DESeq2 (31) and determined the MexT regulon. We defined DEGs as genes in the *mexT* knockout mutant that have at least a 2-fold change compared with wild-type PAO1 and a *P-*value < 0.05. In total, we identified 152 DEGs at an OD_600_ of 1.0, with 104 genes exhibiting higher expression in the *mexT* knockout mutant and 48 genes having higher expression in wild-type PAO1 (Table S1). We identified 265 DEGs at an OD_600_ of 2.0, with 178 genes showing higher expression in the *mexT* knockout mutant and 87 genes having higher expression in wild-type PAO1 (Table S2). Based on this experiment, the MexT regulon size increased during growth. We observed 98 genes shared between the regulon at an OD_600_ of 1.0 and 2.0, demonstrating that over 60% of genes at OD_600_ of 1.0 continued to be expressed and differentially regulated later in growth. As a control (to account for the constitutive expression of MexT in WT PAO1), we also evaluated the transcriptome of a MexS-corrected version of PAO1 (21, 22), which has an intact copy of *mexT* but markedly reduced expression of this gene compared with WT PAO1. We found 150 genes differentially expressed at OD_600_ of 1.0 and 305 and OD_600_ of 2.0 (Tables S3 and S4). This regulon was not substantially different than that of PAO1∆*mexT*, so for subsequent analyses we focused on the MexT regulon.

**Fig 2:**
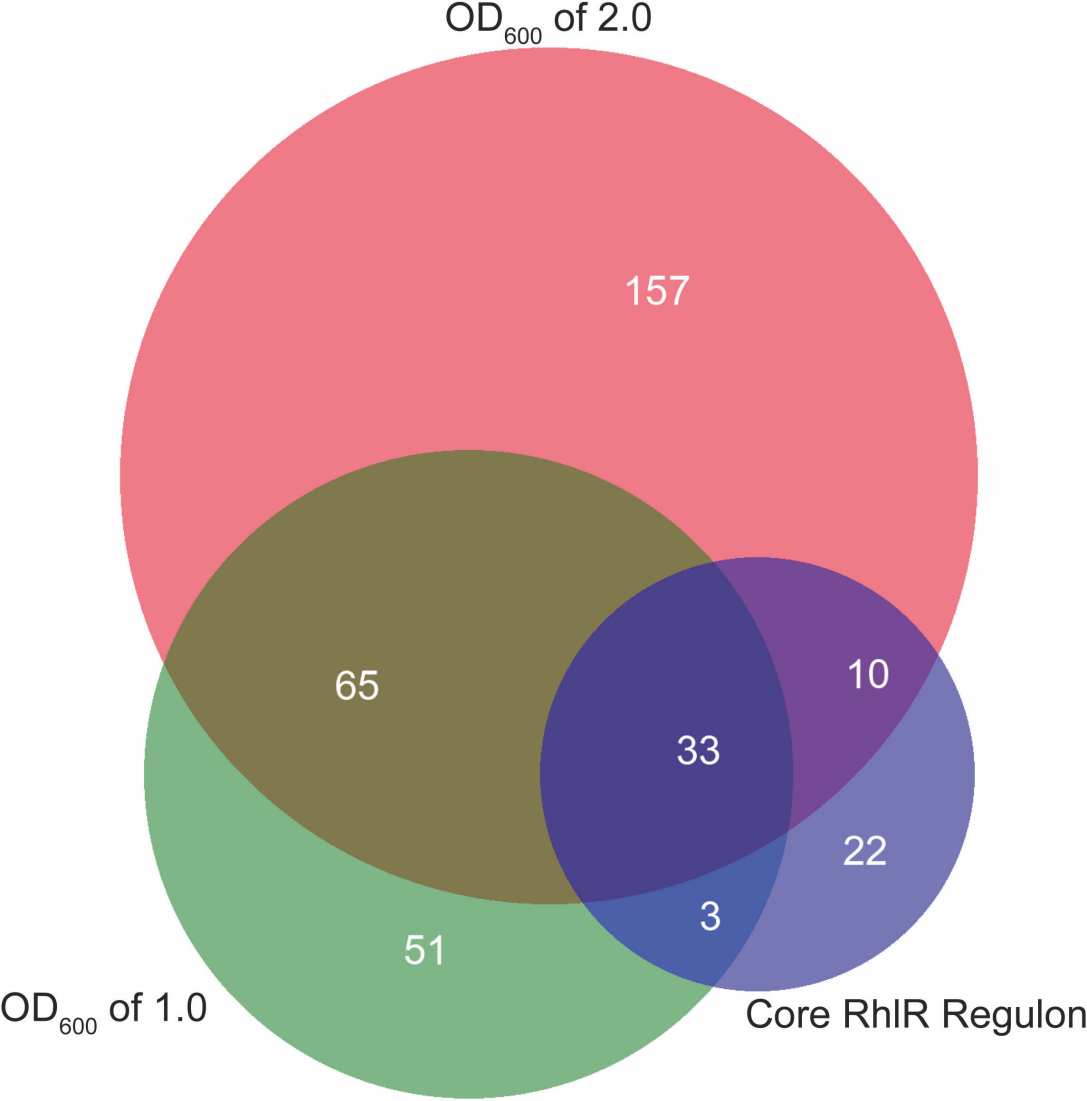
The MexT regulon encompasses at least 150 genes and overlaps with the RhlR regulon. Venn diagram showing the overlap of genes between an OD_600_ of 1.0 and 2.0, and a previously identified RhlR regulon (15, 16, 32).

A PAO1∆*mexT* mutant showed an earlier and higher magnitude of RhlR activation, indicating that MexT function likely negatively affects RhlR activity in wild-type PAO1 (Fig. 1). Consistent with this observation, the transcriptomes we identified included several genes regulated by RhlR. These genes are probably not contributing to MexT-specific regulation of QS. Therefore, we filtered out genes that belong to the core RhlR regulon that was identified previously (using the laboratory strain PAO1 and clinical isolates derived from people with cystic fibrosis) (Fig. 2; Table 1) (15, 16, 32). Because there was robust RhlR activation at both OD_600_ of 1.0 and 2.0, we focused on the OD_600_ 1.0 transcriptome for the remainder of our work, since it was more likely to include factors relevant to RhlR regulation and fewer genes that are activated by RhlR itself.

**TABLE 1:** The MexT regulon: gene expression changes in PAO1**∆***mexT* compared to PAO1^*a*^

Gene	Log_2_ fold change	*P*-value	Gene	Log_2_ fold change	*P*-value	Gene	Log_2_ fold change	*P*-value
PA4881	−6.5	6.71E−30	PA2811	−1.3	9.80E−17	*opdC*	1.3	4.27E−16
*mexF*	−6.4	<1.0E−40	PA2358	−1.3	2.19E−02	*opmD*	1.3	1.46E−04
*mexE*	−6.3	<1.0E−40	*pagL*	−1.2	1.78E−20	*kdpB*	1.3	1.44E−05
PA3229	−6.1	<1.0E−40	PA2812	−1.2	3.53E−07	*pqsA*	1.4	3.63E−15
PA1942	−6.0	<1.0E−40	*nosL*	−1.2	6.82E−04	*stp1*	1.4	3.19E−06
*oprN*	−6.0	<1.0E−40	PA3283	−1.1	9.10E−05	*pqsC*	1.4	1.19E−05
PA4623	−5.8	<1.0E−40	PA2354	−1.1	1.65E−03	*mexH*	1.4	3.65E−07
PA1744	−4.6	<1.0E−40	*bexR*	−1.1	4.11E−12	*ladS*	1.4	5.18E−26
PA2486	−4.5	<1.0E−40	PA1418	−1.1	1.70E−02	*pqsB*	1.4	1.64E−07
PA1743	−4.2	<1.0E−40	*chiC*	1.0	1.19E−03	*pscF*	1.4	1.05E−03
PA3871	−3.8	6.57E−05	PA1658	1.0	3.37E−02	PA1669	1.4	4.88E−11
PA4354	−3.8	<1.0E−40	*pchA*	1.0	8.82E−04	PA1668	1.4	8.81E−07
*narI*	−3.8	5.45E−05	*oprB*	1.0	1.77E−08	*pchE*	1.4	4.11E−12
PA2487	−3.7	<1.0E−40	*phzS*	1.0	5.93E−04	*phzD2*	1.4	5.31E−04
*narJ*	−3.7	3.32E−04	PA2097	1.0	1.21E−02	*phnA*	1.4	7.42E−10
*moaA1*	−3.6	4.17E−05	PA2362	1.1	3.20E−02	*rbsC*	1.5	3.19E−06
PA2485	−3.5	<1.0E−40	PA3377	1.1	2.71E−03	PA2782	1.5	4.88E−05
PA1970	−3.5	<1.0E−40	PA4341	1.1	3.22E−02	PA2068	1.5	1.44E−05
PA3230	−3.2	<1.0E−40	*phzE2*	1.1	3.10E−04	PA3574a	1.5	5.04E−03
PA4355	−2.9	1.10E−29	*bkdA1*	1.1	1.50E−02	PA2067	1.6	3.12E−03
*narH*	−2.7	2.04E−05	*pscE*	1.1	7.13E−03	*hcpA*	1.6	5.93E−06
*gbuA*	−2.6	2.06E−14	PA4219	1.1	1.01E−03	PA2781	1.6	4.08E−08
PA1333	−2.6	1.20E−24	*kdpC*	1.1	3.09E−02	*rbsA*	1.6	1.21E−03
PA2813	−2.5	<1.0E−40	PA3677	1.1	1.19E−03	PA4142	1.7	3.10E−14
*xenB*	−2.4	3.19E−21	*stk1*	1.2	1.09E−06	*hcpB*	1.7	5.57E−06
*nosY*	−2.2	2.17E−04	PA1657	1.2	5.06E−03	*pchF*	1.7	5.28E−04
PA2491	−2.2	<1.0E−40	*pchD*	1.2	4.78E−04	PA2260	1.8	1.49E−07
PA1419	−2.1	1.04E−09	*phzF2*	1.2	1.57E−03	*pchC*	1.8	1.49E−07
*nirN*	−2.1	5.30E−06	*pqsD*	1.2	1.05E−04	*hisJ*	1.8	1.55E−02
PA0510	−2.1	1.17E−08	*hcpC*	1.2	1.42E−03	*pchB*	1.8	1.48E−07
PA2759	−2.0	2.58E−30	PA0050	1.2	6.64E−05	PA3519	1.8	7.49E−03
PA4882	−2.0	7.54E−35	PA2322	1.2	1.09E−03	PA4596	1.8	1.56E−02
PA1420	−1.9	1.30E−14	*popN*	1.3	3.56E−04	PA2069	1.8	1.18E−11
PA3381	−1.7	5.26E−03	*bkdA2*	1.3	5.90E−10	*oprD*	1.9	2.63E−27
PA1417	−1.6	3.35E−06	*hpcG*	1.3	1.58E−12	PA3325	2.0	1.37E−25
PA2758	−1.6	5.48E−22	PA5180	1.3	1.19E−03	PA5328	2.2	1.60E−02
PA3282	−1.5	3.62E−04	*pqsE*	1.3	1.19E−05	*pcrG*	2.2	1.28E−04
PA1416	−1.5	5.77E−06	PA4218	1.3	2.23E−05	PA0165	2.3	7.90E−33
						PA4133	2.7	1.12E−27

^*a*^ We performed RNA-seq on wild-type PAO1 and PAO1∆*mexT* grown to an OD_600_ of 1.0. Differentially expressed genes are defined as genes that showed a 2-fold change compared to wild-type PAO1 and had a *P*-value less than 0.05. We further filtered the differentially expressed genes to exclude those that belong to the core RhlR regulon.

Using the OD_600_ 1.0 transcriptome, we identified 115 genes that belong solely to the MexT regulon, with 47 genes that appear to be activated by MexT (genes with lower expression in PAO1∆*mexT* compared to wild-type PAO1) and 68 genes that appear to be repressed (genes with higher expression in PAO1∆*mexT* compared with wild-type PAO1). Within the MexT regulon, we identified 51 genes that were identified in the previous study that used a MexT overexpression mutant (28). In total, over 30% of the genes identified in the prior study were identified in ours. The genes we identified in the MexT regulon at an OD_600_ of 1.0 belong to several protein families, including transporters, phenazine biosynthesis, and transcriptional regulators.

To identify MexT effectors that potentially modulate QS, we filtered for genes that have a high confidence, such as those exhibiting large fold changes, identification of whole operons, and genes with known QS interactions. Our data validate other studies showing that MexT is a transcriptional regulator of the multidrug RND efflux pump MexEF-OprN (29, 33). MexEF-OprN is known for the export of the quorum-sensing signal HHQ, a PQS precursor molecule, resulting in delayed PQS QS (27). A recent study screening for QS-inhibitory compounds identified Z-ethylthioenynone as a molecule that targets MexEF-OprN and affects C4-HSL concentrations intra- and extracellularly in wild-type PAO1 (34). Given these facts, it is plausible that MexEF-OprN expression is induced by constitutive MexT activity and modulates QS architecture by altering QS signal concentrations and affecting QS activation.

### MexT regulation of efflux pumps affects the QS hierarchy

We explored the role of MexEF-OprN in modulating QS in PAO1. We hypothesized that, like MexT, MexEF-OprN negatively regulates RhlR activity. To test this hypothesis, we first made a *mexEF* knockout mutant (PAO1∆*mexEF*) to disrupt the efflux activity of MexEF-OprN. Like other RND systems found in *P. aeruginosa*, membrane transport of substrates requires all three proteins to function (35).

**Fig 3:**
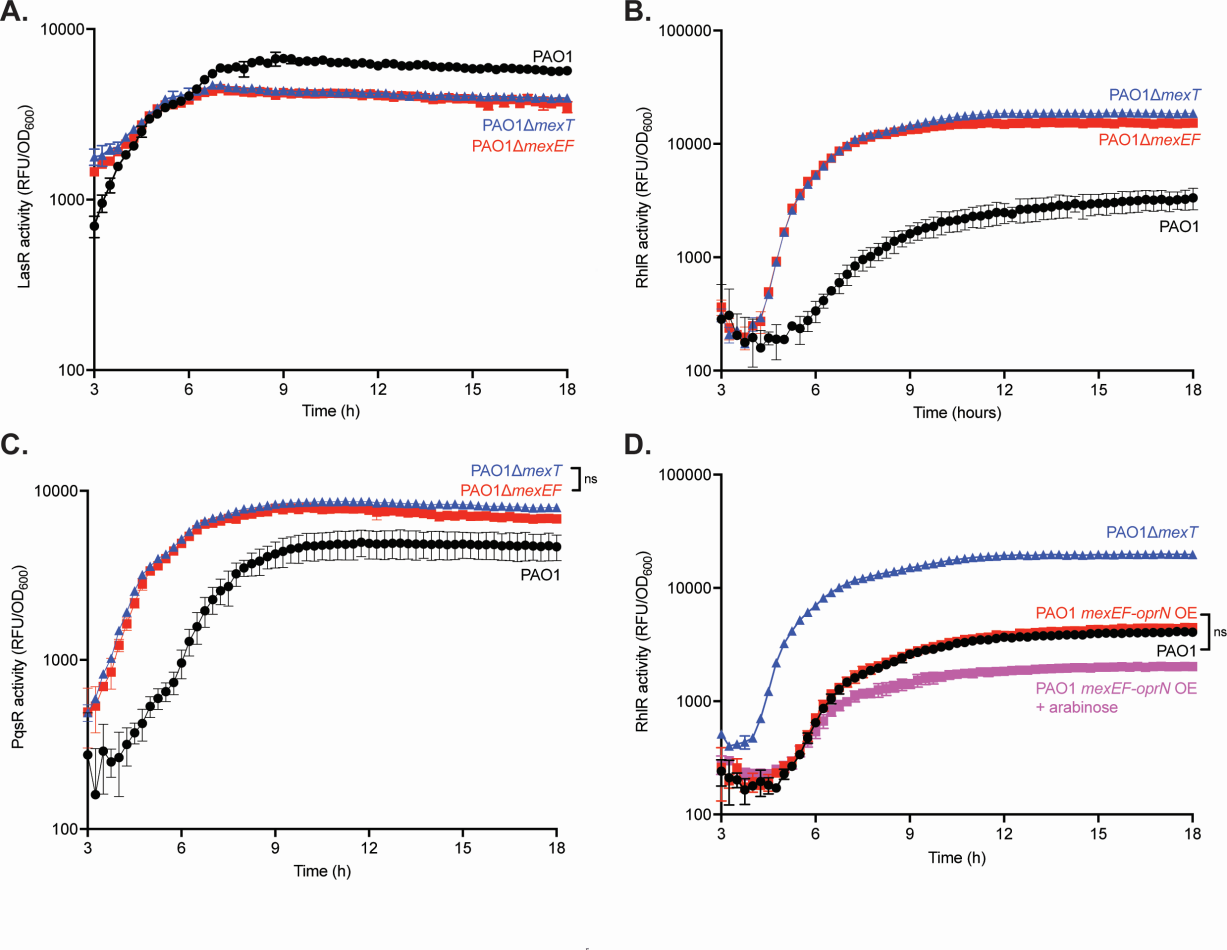
MexEF-OprN influences both RhlR and PqsR QS activity. (**A**). The LasR activity of PAO1, PAO1∆*mexT*, and PAO1∆*mexEF* was determined using a LasR activity (*lasI-gfp*) reporter plasmid. (**B**). RhlR activity for PAO1, PAO1∆*mexT*, and PAO1∆*mexEF* was determined using a RhlR activity (*rhlA-gfp*) reporter plasmid. (**C**). PqsR activity for PAO1, PAO1∆*mexT*, and PAO1∆*mexEF* was determined using a PqsR activity (*pqsA-gfp*) reporter plasmid. (**D**). RhlR activity for PAO1, PAO1∆*mexT*, PAO1∆*mexEF*, and PAO1 + *mexEF* oprN OE was determined using a RhlR activity reporter plasmid. Each experiment was performed in triplicate. Error bars represent the standard deviation. *P* values were calculated using a two-way ANOVA with Geisser-Greenhouse correction, where all strains were compared with wild-type PAO1. All unannotated comparisons met a *P* < 0.05, and non-significant comparisons are annotated with “ns.”

We then introduced episomal plasmids with reporters for LasR (*lasI-gfp*), RhlR (*rhlA-gfp*), or PqsR (*pqsA-gfp*) activity. The *mexEF* knockout mutant shows similar LasR activity and timing compared with wild-type PAO1 and the *mexT* knockout mutant (Fig. 3A). However, RhlR activity was different: we observed that the *mexEF* knockout mutant, like PAO1∆*mexT*, showed earlier and higher RhlR activity compared with wild-type PAO1 (Fig. 3B). We tested whether this change in RhlR activity was in fact mediated by MexT by generating a *mexT* complement, which harbors a copy of *mexT* and its endogenous promoter at the chromosomal *att* site (Fig. S1). Restoration of *mexT* decreased RhlR activity similar to that observed in wild-type PAO1. This finding demonstrates that MexEF-OprN is an important MexT-regulated efflux pump that modulates QS. We also found PqsR activity earlier in both the *mexT* and *mexEF* knockout mutants (Fig. 3C). In parallel, we tested the role of MexEF-OprN in regulating RhlR QS by creating an integrating plasmid that contains *mexEF-oprN* under the control of an arabinose-inducible promoter (PAO1 + *mexEF oprN* OE) and compared this mutant with wild-type and PAO1∆*mexT* (Fig. 3D). When we induced expression of *mexEF-oprN,* we found that the pump alters the magnitude, but not the timing, of RhlR activation compared with PAO1 and PAO1 *mexEF-oprN* OE without arabinose. These results corroborate the idea that MexEF-OprN activity impacts RhlR QS. Together, they demonstrate that both *mexT* and *mexEF* knockout mutants affect RhlR and PqsR QS. Because PAO1∆*mexEF* phenocopies PAO1∆*mexT*, it shows that MexT regulation of RhlR QS acts at least in part through the efflux pump MexEF-OprN, indicating a pathway from MexT to MexEF-OprN to RhlR QS.

**Fig 4:**
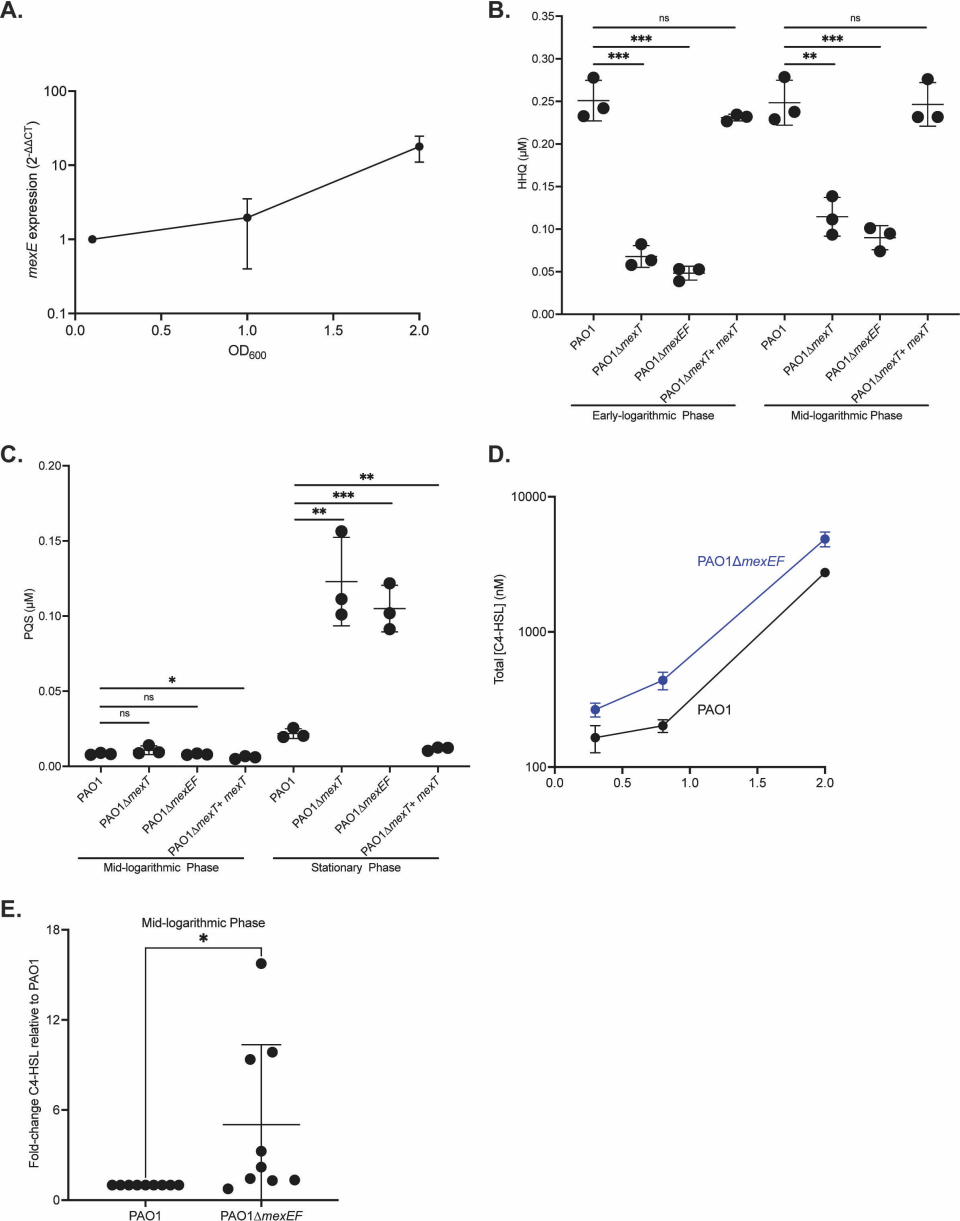
Expression of *mexEF-oprN* does not account for changes in RhlR activity, but signal concentrations differ between strains. (**A**). Quantitative real-time PCR measuring *mexE* transcripts during bacterial growth of wild-type PAO1. *mexE* expression was normalized to *rplU* and reported as 2^-∆∆CT^. (**B and C**). HHQ (**B**) and PQS (**C**) were extracted from the total cell culture during various growth phases and measured by LCMS. *P* values were calculated using an unpaired two-tailed *t* test with Welch’s correction. * denotes a *P* < 0.05, ** denotes a *P* < 0.01, and *** denotes a *P* < 0.001. (**D**). C4-HSL was extracted from the total cultures during bacterial growth. C4-HSL concentrations were determined using a biological reporter assay. *P* values were calculated at each growth phase using an unpaired two-tailed *t* test with Welch’s correction. All comparisons had a *P* < 0.0001. (**E**). C4-HSL was extracted from the intracellular fraction during bacterial growth. C4-HSL concentrations in the cellular fraction were determined using the biological reporter assay. Since the determination of cell volume is not possible, the concentrations were normalized to WT PAO1. *P* values were calculated using an unpaired two-tailed *t*-test with Welch’s correction. * denotes a *P* < 0.05. Each experiment was performed in triplicate. Error bars represent the standard deviation.

Although RhlR activity is negatively influenced by MexEF-OprN and MexT in wild-type PAO1, RhlR activity eventually occurs during later growth phases, indicating that either the negative regulation is relaxed or a positive regulatory pathway is induced. To determine whether *mexEF-oprN* expression decreases over time and leads to the increase in RhlR activity, we performed a quantitative real-time PCR experiment to monitor *mexE* expression over bacterial growth, with each subsequent time point compared with time 0 (Fig. 4A). We observed that *mexE* expression slightly increased at an OD_600_ of 1.0 and then further increased by more than 10-fold at an OD_600_ of 2.0. These results show that the increase in RhlR activity in PAO1 at later time points is not driven by a reduction in *mexEF-oprN* expression.

We also tested whether the delay in RhlR activity in wild-type PAO1 was due to MexEF-OprN modulating production of QS signals. HHQ is a substrate of the MexEF-OprN pump (27). We observed higher total concentrations of HHQ in wild-type PAO1 compared with both the PAO1∆*mexT* and PAO1∆*mexEF* mutants (Fig. 4B). This finding is consistent with the idea that the MexEF-OprN pump maintains higher extracellular HHQ concentrations, which leads to less conversion to PQS by the intracellular PqsH enzyme. We also assessed whether overall PQS concentrations are altered in the PAO1∆*mexT* and PAO1∆*mexEF* mutants. We observed that PQS concentrations were similar between wild-type PAO1 and both mutants at mid-logarithmic phase, but there was an approximately 10-fold increase in PQS concentrations in both mutants compared with wild-type PAO1 in stationary phase (Fig. 4C). Although the timing of the increase in PQS concentrations does not align precisely with the increase in RhlR activity, the increase in PQS concentrations indicates heightened PQS QS.

We next assessed whether C4-HSL concentrations are altered by MexEF-OprN. If C4-HSL is a substrate of MexEF-OprN, and MexEF-OprN delays RhlR QS by exporting C4-HSL signal, then we would expect a lower intracellular concentration of C4-HSL in wild-type PAO1 compared with the PAO1∆*mexEF* mutant. We tested this hypothesis by measuring total and intracellular C4-HSL concentrations in wild-type PAO1 and PAO1∆*mexEF* over time (Fig. 4D and E). We observed that total concentrations of C4-HSL were higher at all growth phases for the PAO1∆*mexEF* mutant compared with wild-type PAO1. This is consistent with the earlier and higher RhlR activity observed in both the PAO1∆*mexT* and PAO1∆*mexEF* mutants (Fig. 3). Although residual extracellular PBS from washes dilutes intracellular C4-HSL measurements, we can compare the intracellular C4-HSL concentrations in mutants relative to wild-type PAO1. We observed 5-fold higher intracellular C4-HSL concentrations in the PAO1∆*mexEF* compared with wild-type PAO1 at mid-logarithmic growth phase (Fig. 4E). This result is consistent with the premise that in wild-type PAO1, MexEF-OprN exports C4-HSL, leading to lower intracellular C4-HSL, thus delaying RhlR QS. Together, these experiments show that MexEF-OprN alters both PQS and C4-HSL signaling in a way that negatively affects RhlR activity.

### MexT regulation of *pqsE* also contributes to the QS hierarchy

MexEF-OprN delays PQS QS through binding and exporting the PQS precursor HHQ (27). The gene product of *pqsE*, which is regulated by PQS QS, is known to interface with Rhl QS by positively regulating activation of some genes in the RhlR regulon (36, 37). We observed that the MexT regulon at an OD_600_ of 1.0 included the entire PQS biosynthesis operon *pqsABCDE,* which includes the gene encoding the chaperone-like PqsE protein. We also measured an increase in PQS concentrations at later growth phases, which is consistent with increased PQS QS (Fig. 4C). Because we identified these PQS biosynthesis genes in our RNA-seq experiment and have shown a role of MexEF-OprN in regulating RhlR QS, we hypothesized that constitutive MexT activity induces *mexEF-oprN* expression, leading to the repression of PQS QS, delayed *pqsE* expression, and delayed RhlR activation.

**Fig 5:**
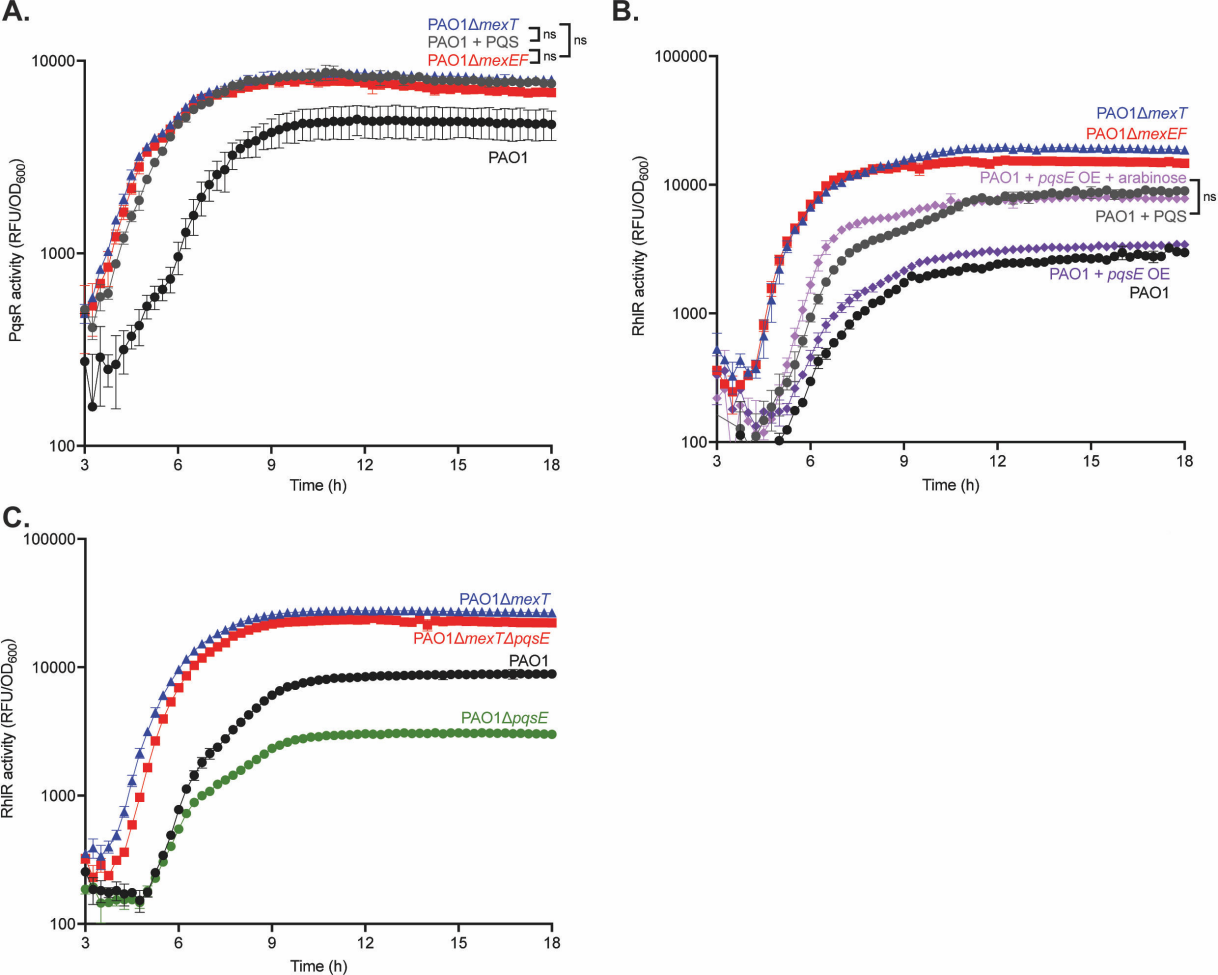
PqsE partially regulates RhlR activity. (**A**). PqsR activity with and without the addition of 20 µM PQS for PAO1, PAO1∆*mexT*, and PAO1∆*mexEF* was determined using a PqsR activity reporter plasmid. (**B**). RhlR activity with and without the addition of 20 µM PQS for PAO1, PAO1∆*mexT*, PAO1∆*mexEF*, and PAO1 + *pqsE* with and without arabinose was determined using a RhlR activity reporter plasmid. (**C**). RhlR activity for PAO1, PAO1∆*mexT*, and PAO1∆*mexT*∆*pqsE* was determined using a RhlR activity reporter plasmid. Each experiment was performed in triplicate. Error bars represent the standard deviation. *P* values were calculated using a two-way ANOVA with Geisser-Greenhouse correction, where all strains were compared with wild-type PAO1. All unannotated comparisons met a *P* < 0.05, and non-significant comparisons are annotated with “ns.”

We tested whether MexT delays PQS QS through the MexEF-OprN efflux pump. We measured PqsR activity in wild-type PAO1 supplemented with exogenous PQS and compared that with the PAO1∆*mexT* and PAO1∆*mexEF* mutants (Fig. 5A). We found that the addition of exogenous PQS to the wild-type culture phenocopies the PqsR activity we observed in both PAO1∆*mexT* and PAO1∆*mexEF*, demonstrating that the delay in PQS activity in wild-type PAO1 can be attributed to the regulatory activity of MexT and MexEF-OprN.

We next determined whether this delay in PQS QS affected RhlR QS. We measured RhlR activity in PAO1∆*mexT* and PAO1∆*mexEF*, and we reasoned that the addition of exogenous PQS would alter RhlR activity to match that of the PAO1∆*mexT* and PAO1∆*mexEF* mutants, like PqsR activity (Fig. 5B). We found that the addition of exogenous PQS did indeed result in earlier and higher RhlR activity compared with wild-type PAO1. However, the activity was intermediate between wild-type PAO1 and both PAO1∆*mexT* and PAO1∆*mexEF*, indicating that the delay in PQS QS only partially accounts for the relative delay of RhlR QS in wild-type PAO1.

Because we observed a change in RhlR activity with the addition of PQS, we sought to identify the factor mediating this change. PqsE has a chaperone-like role, stabilizing RhlR and increasing its affinity for target promoters. Furthermore, based on our transcriptome data, we observed higher expression of *pqsE* in the PAO1∆*mexT* mutant compared with wild-type PAO1. Together, these data led us to speculate that the regulation of RhlR activity in wild-type PAO1 was due to repression of *pqsE*. To test this hypothesis, we constructed a mutant to counter *pqsE* repression mediated by MexT. This mutant contains an integrated plasmid with *pqsE* under the control of an arabinose-inducible promoter (PAO1 + *pqsE* OE), and we compared RhlR activity in this mutant with wild-type PAO1, PAO1∆*mexT*, and PAO1∆*mexEF* (Fig. 5B). We found that PAO1 + *pqsE* OE without arabinose showed similar RhlR activity as wild-type PAO1, indicating negligible basal expression from the promoter. However, with the addition of arabinose, PAO1 + *pqsE* OE showed nearly identical RhlR activity to wild-type supplemented with exogenous PQS.

We further assessed the role of PqsE by creating a *mexT* and *pqsE* double knockout mutant (PAO1∆*mexT*∆*pqsE*) and compared RhlR activity in this mutant with wild-type PAO1 and PAO1∆*mexT* (Fig. 5C). In agreement with the prior experiment, we observed a decrease in RhlR activity that occurred at a slightly later time point in PAO1∆*mexT*∆*pqsE* compared with PAO1∆*mexT*. However, PAO1∆*mexT*∆*pqsE* retained higher RhlR activity compared with PAO1, indicating that *pqsE* is necessary but not sufficient for MexT- and MexEF-OprN-mediated regulation of RhlR activity. These experiments showed that the increase in RhlR activity due to the addition of PQS is likely mediated by the earlier activation of PQS QS and increased production of PqsE.

### MexT regulation of MexEF-OprN and PqsE affects virulence

**Fig 6:**
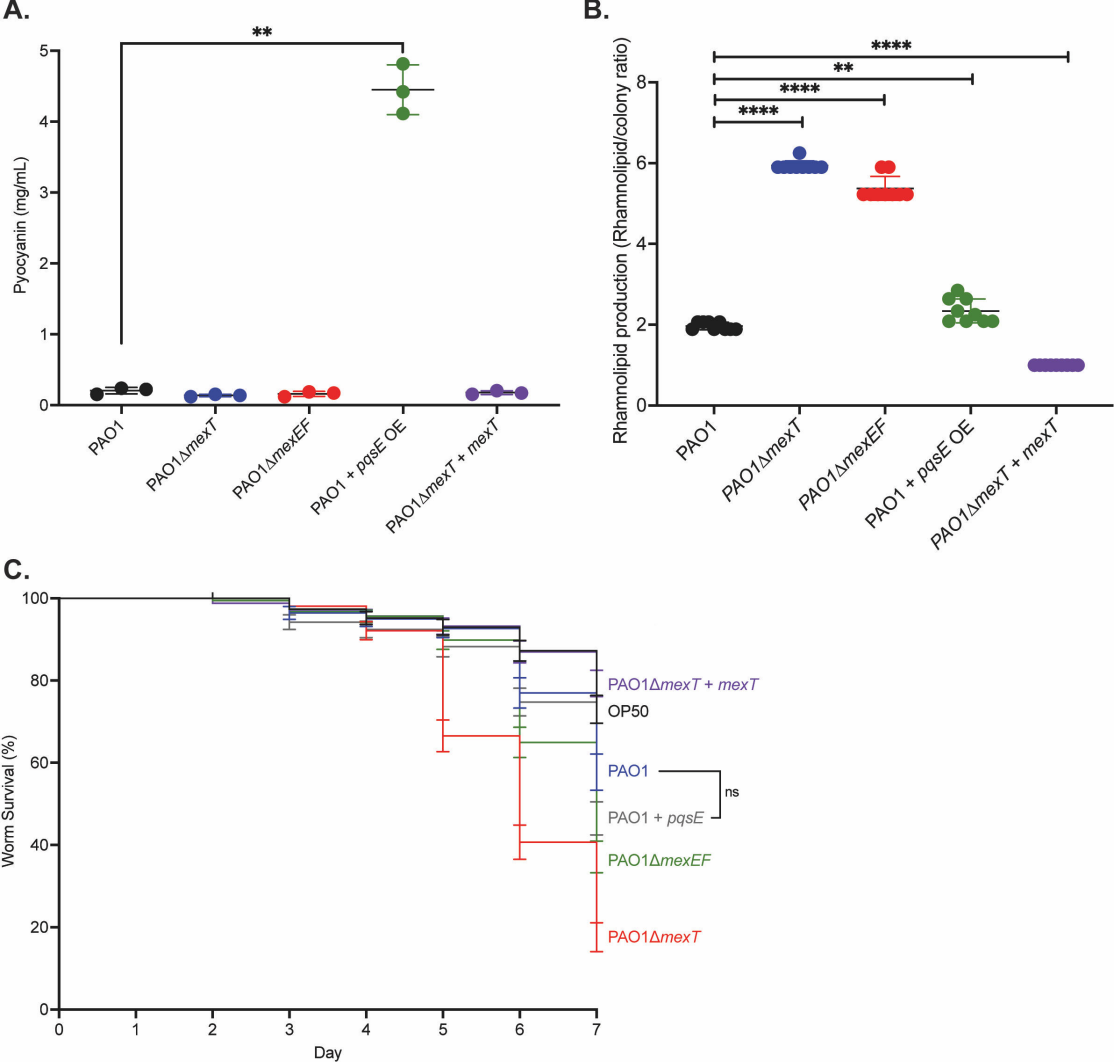
MexEF-OprN and PqsE affect virulence. (**A**). Pyocyanin production after 24 h of growth was measured in wild-type PAO1 (black), PAO1∆*mexT* (blue), PAO1∆*mexEF* (red), PAO1 + *pqsE* (green), and PAO1∆*mexT + mexT* (purple). Error bars represent the standard deviation. *P* values were calculated using an unpaired two-tailed *t*-test with Welch’s correction, where all strains were compared with wild-type PAO1. ** denotes a *P* < 0.01. (**B**). Rhamnolipid production was quantified by measuring the halo area (mm^2^) produced using methylene blue-containing plates. Graphs show the ratio of the halo area to the area of the bacterial colony. Error bars represent the standard deviation. *P* values were calculated using an unpaired two-tailed *t*-test with Welch’s correction, where all strains were compared to wild-type PAO1. **** denotes a *P* < 0.0001. (**C**). Survival of *C. elegans* when challenged with PAO1 and various mutants. Data are representative of three experiments, and error bars represent standard error. *P* values were calculated using a Gehan-Breslow-Wilcoxon test, where all strains were compared with wild-type PAO1. All unannotated comparisons met a *P* < 0.01, and non-significant comparisons are annotated with “ns.” Each experiment was performed in triplicate.

QS regulates virulence functions (3); hence, we next assessed the role of the MexT-regulated genes on virulence. We observed that expression of *pqsE* significantly increases the production of pyocyanin compared with wild-type PAO1 and aligns with known roles of PqsE in regulating pyocyanin production (38). We found that pyocyanin production in all other mutants was unchanged compared with wild-type PAO1 (Fig. 6A). We next examined production of rhamnolipids, which are RhlR-regulated biosurfactants. We found that PAO1∆*mexT*, PAO1∆*mexEF*, and PAO1 + *pqsE* OE mutants all exhibited more rhamnolipid production than wild-type PAO1 (Fig. 6B). Furthermore, we found that *mexT* complementation of PAO1∆*mexT* restored rhamnolipid production to wild-type levels. These data are consistent with MexT, PqsE, and MexEF-OprN influencing some facets of virulence. Finally, we tested these mutants in an assay to assess virulence more broadly, specifically using a *C. elegans* virulence assay (Fig. 6C). Both *mexT* and *mexEF* mutants showed a significant increase in worm mortality compared with wild-type PAO1. The *pqsE* overexpression mutant showed a non-significant increase in worm mortality, indicating that PqsE has a smaller role in overall virulence in this model. We found that *mexT* complementation resulted in increased worm survival. Overall, MexT and MexEF-OprN, and to a lesser extent PqsE, impact QS in a way that ultimately affects *Pa* pathogenicity.

**Fig 7:**
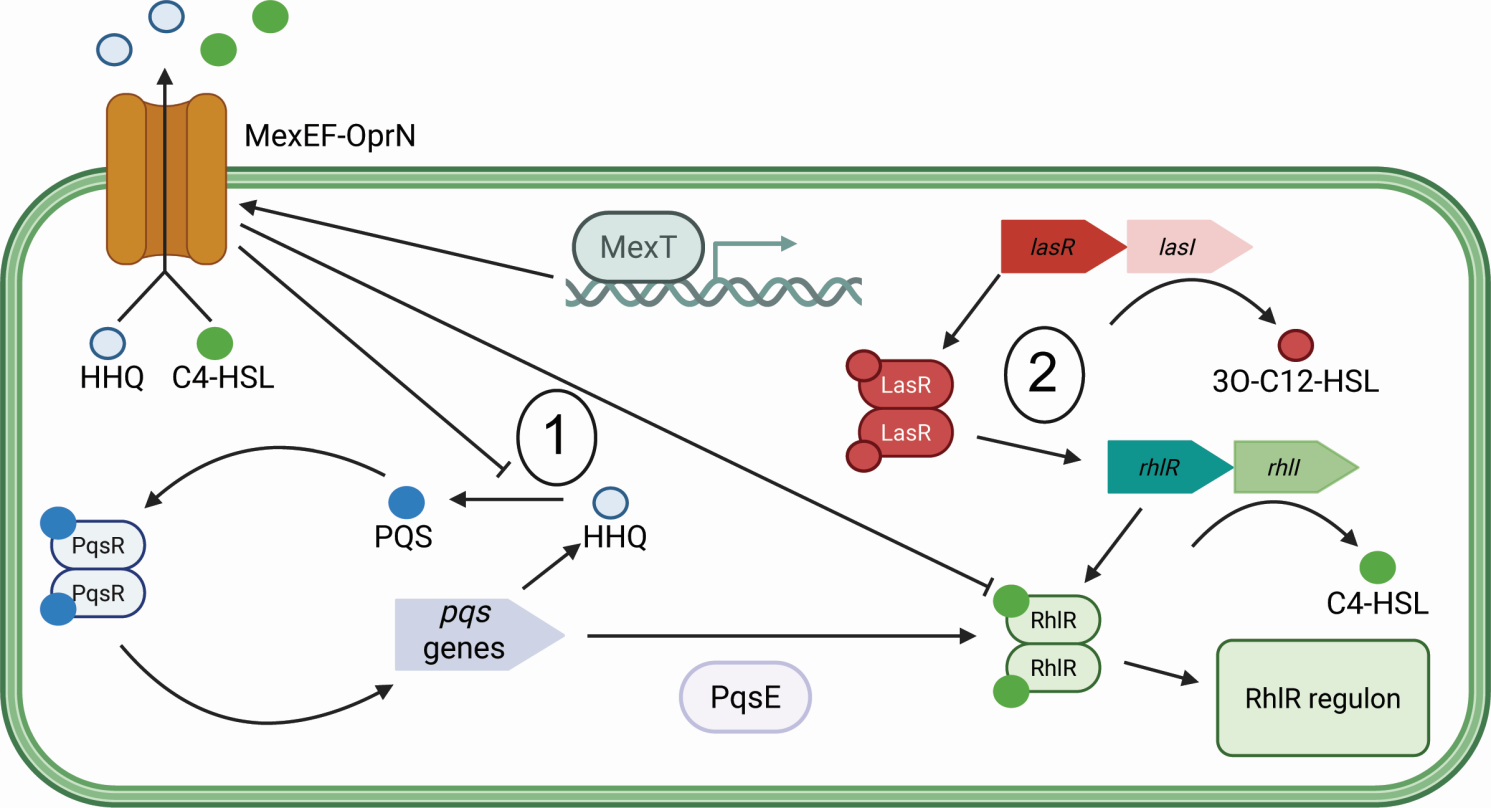
MexT negatively influences RhlR activity, whereas LasR positively regulates *rhlR* expression. Cartoon showing the model of proposed MexT-mediated regulation of RhlR. At lower cell density (denoted with “1”), MexT positively regulates *mexEF-oprN*, delaying RhlR QS through decreasing intracellular C4-HSL. In parallel, MexEF-OprN exports the signal HHQ, delaying the activation of PQS QS and *pqsE* expression. Both events result in less active RhlR. At higher cell densities (denoted with “2”), LasR activity positively regulates *rhlR* expression and an increase in RhlR activity.

Together, these data enhance prior work on MexT, MexEF-OprN, and RhlR QS (Fig. 7). We showed that constitutive MexT activity in PAO1 resulted in increased expression of efflux pump genes *mexEF-oprN*. At lower cell densities, the MexEF-OprN activity repressed RhlR activity through two mechanisms. This is partially mediated by the delay in both PQS activation and the production of PqsE that would normally activate RhlR. MexEF-OprN also directly delays RhlR activation, likely by reducing intracellular C4-HSL concentrations. There is also an unidentified product influenced by MexEF-OprN that further represses RhlR activity. At higher cell densities, LasR activity counteracts the repression by MexEF-OprN and results in *rhlI/rhlR* and *pqsR* expression, leading to RhlR activity.

### MexT is not the sole factor affecting QS architectures in *P. aeruginosa*

We wondered about the diversity of QS architectures and their regulation in other *P. aeruginosa* isolates. Recent work has identified clinical and environmental isolates that exhibit LasR-independent RhlR QS, but these had *lasR* null mutations at the time of isolation. It is possible that other mutations (such as in *mexT*) resulted in LasR-independent RhlR QS in these isolates. To probe QS architecture, we queried whether the LasR-dependent hierarchy was present in environmental or clinical isolates. We reasoned that if we knock out *lasR* in these isolates and RhlR activity is maintained, then these isolates encode a QS architecture different from PAO1. However, if we knock out *lasR* in these isolates and RhlR activity is abrogated, then the isolates display QS architecture akin to PAO1.

**Fig 8:**
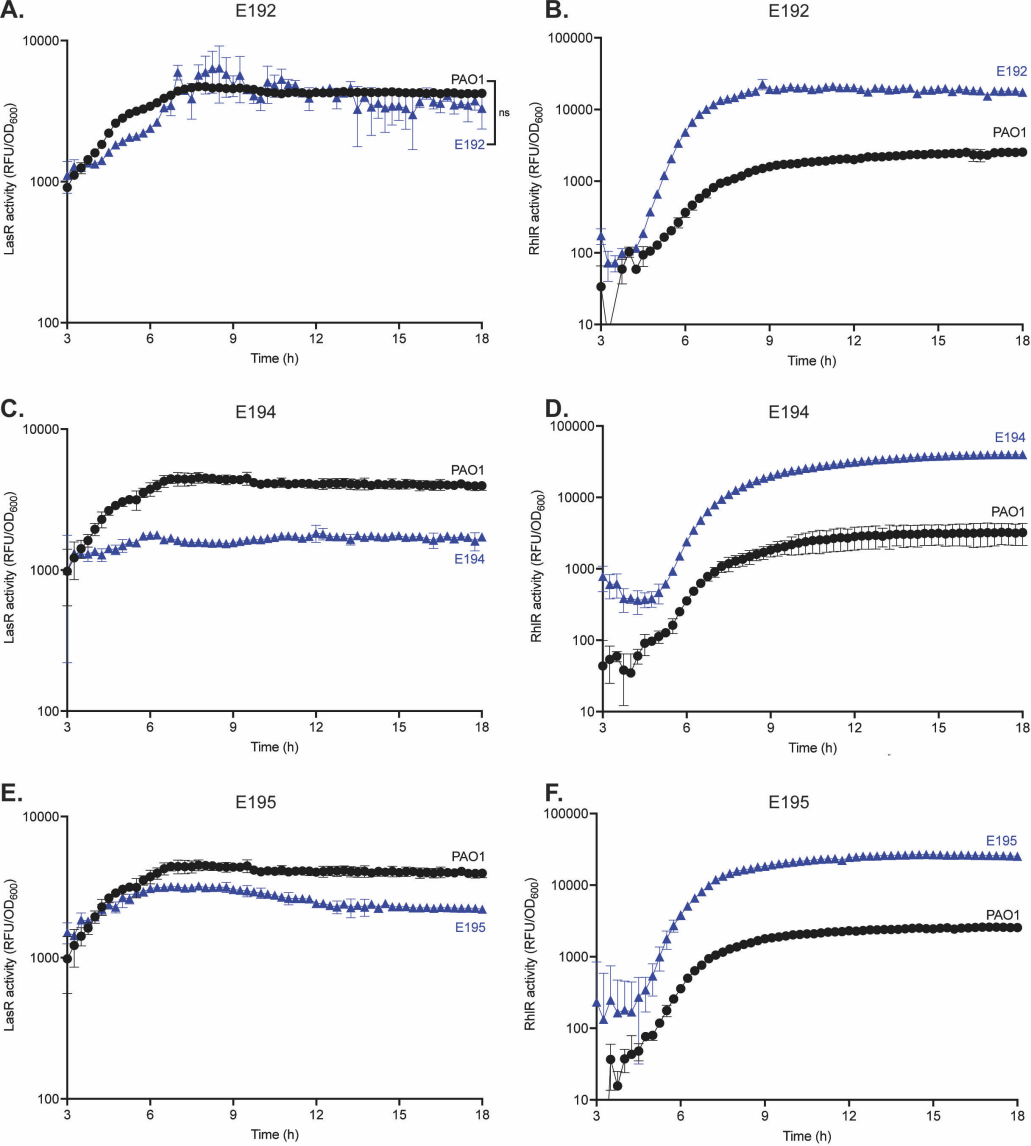
RhlR, but not LasR, differs between PAO1 and EPIC isolates. LasR activity for PAO1 compared to E192 (**A**), E194 (**C**), and E195 (**E**),was measured using a LasR activity reporter plasmid. RhlR activity for PAO1 compared with E192 (**B**), E194 (**D**), and E195 (**F**) was measured using a RhlR activity reporter plasmid. Each experiment was performed in triplicate. Error bars represent the standard deviation. *P* values were calculated using a two-way ANOVA with Geisser-Greenhouse correction, where all strains were compared to wild-type PAO1. All unannotated comparisons met a *P* < 0.05, and non-significant comparisons are annotated with “ns.”

We started by assessing LasR and RhlR kinetics in three clinical isolates from chronic infections in people with cystic fibrosis (E192, E194, and E195) (17). These are isolates from the Early *Pseudomonas* Infection Control (EPIC) study (39) and harbor functional LasR proteins. Each isolate was transformed with the LasR and RhlR reporter plasmids described earlier and compared to wild-type PAO1 (Fig. 8). All clinical isolates had active LasR QS that was similar to PAO1. In contrast, RhlR activity occurred earlier in all clinical isolates compared with PAO1. All three isolates exhibited at least 5-fold higher RhlR activity compared with PAO1. Together, these data show that the clinical isolates exhibit differences in QS compared with PAO1.

**Fig 9:**
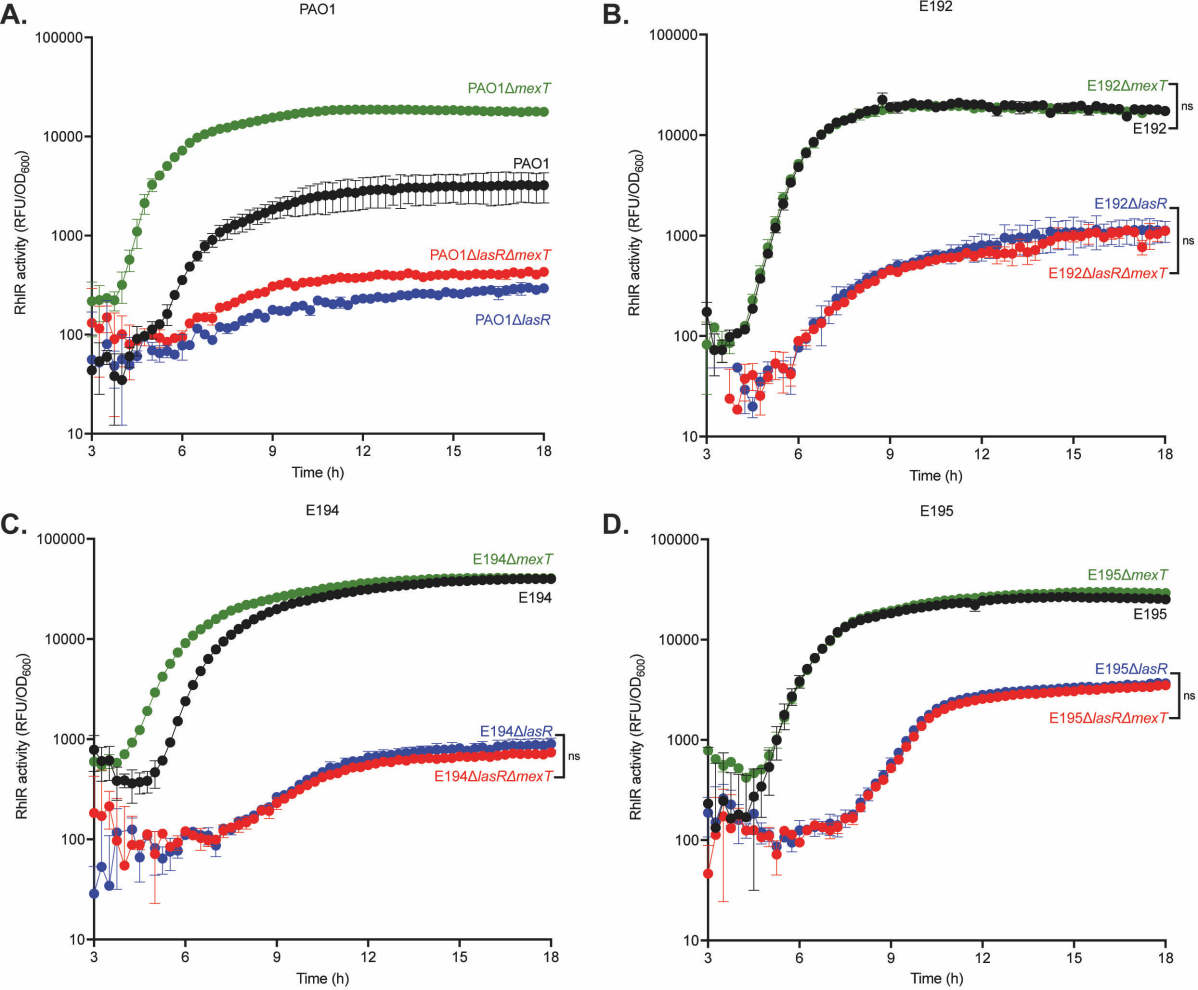
EPIC isolates exhibit LasR-dependent but MexT-independent RhlR activity. RhlR activity for wild-type, *lasR* and *mexT* knockouts*,* and *lasR-mexT* double knockout mutants for PAO1 (**A**), E192 (**B**), E194 (**C**), and E195 (**D**) were measured using a RhlR activity reporter plasmid. Each experiment was performed in triplicate. Error bars represent the standard deviation. *P* values were calculated using a two-way ANOVA with Geisser-Greenhouse correction, where all strains were compared to wild-type PAO1. All unannotated comparisons met a *P* < 0.05, and non-significant comparisons are annotated with “ns.”

We compared the QS architecture of clinical isolates to that of PAO1 by generating *lasR* deletion mutants and measuring RhlR activity (Fig. 9). In all clinical isolates, RhlR activity occurred later and at a lower magnitude with *lasR* deletion compared with the parent (Fig. 9B through D). However, clinical isolates still retained some RhlR activity, which differs from PAO1. Of the three isolates, E195∆*lasR* retained the most RhlR activity. For isolates E192 and E194, RhlR activation in *lasR* mutants followed a linear pattern. For isolate E195, we observed RhlR activation in the *lasR* knockout, followed by an S-curve pattern similar to wild-type PAO1 and the parental strains.

We next assessed whether the variability of LasR dependence was due to MexT modulation of QS architecture. To ask this question, we performed whole genome sequencing on each clinical isolate to identify changes in the overall genome compared with PAO1. Each isolate encodes a larger genome than wild-type PAO1, ranging from 50 kbp to 600 kbp larger. E194 and E195 have identical *mexT* sequences to PAO1. E192 contained non-synonymous mutations that resulted in an I298F and D302E variant. We also assessed the MexT regulator, MexS, which is shown to be inactive in PAO1 due to an N249D mutation. In contrast to PAO1, all of the clinical isolates we studied encode N249, indicating each has a functional MexS protein. When we surveyed a collection of 66 clinical strains for constitutive expression of MexEF-OprN, we found that around 40% had a *nfxC*-like phenotype, as shown by the ability to readily grow on high concentrations of chloramphenicol. The same proportion was obtained with strains isolated from environments other than patients. This finding reveals that PAO1 is not an outlier regarding the overexpression of the MexEF-OprN pump.

To investigate MexT activity in the clinical isolates, we transformed each isolate with a MexT reporter that contains the promoter of *mexE*, cloned upstream of *gfp* in an episomal plasmid. Compared with wild-type PAO1, MexT activity in each clinical isolate was almost undetectable (Fig. S2). MexT is activated due to oxidative stress, and others have demonstrated that the chemical compound diamide induces oxidative stress and activates MexT (24). PAO1 has constitutive MexT activity, but we still observed a slight increase in MexT activity with the addition of diamide. In contrast, with the addition of diamide to each clinical isolate, we observed activation of MexT, indicating that MexT in each clinical isolate is functional, including the double variant encoded in E192.

We next determined if RhlR activity in the clinical isolates was modulated by MexT (as it is in PAO1) by generating *mexT* knockout mutants in each isolate and measuring RhlR activity (Fig. 9). E192∆*mexT* and E195∆*mexT* displayed no differences in RhlR activity compared with wild-type isolates. Interestingly, E194∆*mexT* showed earlier RhlR activity with the same terminal intensity compared with wild-type E194. Altogether, the clinical isolates differ from PAO1: MexT does not substantially change RhlR activity in *mexT* knockout mutants.

We asked whether MexT affects the regulatory relationship between LasR and RhlR QS. In PAO1, we observed an increase in RhlR QS in PAO1∆*lasR*∆*mexT* compared with PAO1∆*lasR* (Fig. 9A). In contrast, each clinical isolate exhibited no change in RhlR activity when comparing *lasR* knockout mutants to *lasR* and *mexT* double knockout mutants (Fig. 9B through D). Together, these results indicate that although the clinical isolates depend on LasR for RhlR activity, as does strain PA14 (24, 40), their RhlR activity is not negatively regulated by MexT as it is in PAO1.

## DISCUSSION

In this study, we explored QS architectures in laboratory and clinical isolates of *P. aeruginosa* and how this architecture is modulated. Our prior work (24, 25) defined the role of MexT in altering the QS hierarchy in *P. aeruginosa* PAO1 and implicated MexT in the modulation of virulence functions. The current work builds on those studies by identifying the MexT-regulated gene products that modulate QS, the influences of these factors on quorum sensing, and the roles of these effectors in virulence factor production and pathogenicity. Through bacterial genetics and transcriptomics, we investigated how the transcription factor MexT promotes a LasR-dependent QS architecture in the laboratory strain PAO1. We discovered that the MexT-regulated genes *mexE*, *mexF*, and *oprN* contribute to QS function. When these are inactivated, there is an increase in RhlR activity compared with the wild-type. MexEF-OprN enforces this architecture through its influence on PQS QS and, by extension, PqsE, which increases RhlR activity. By assessing the diversity of QS architecture across *P. aeruginosa*, we discovered that the QS hierarchy in a subset of clinical isolates from chronic infections of people with cystic fibrosis is LasR-dependent, but the degree of dependence varies across isolates, with none exhibiting absolute LasR dependence. Finally, we found that although there is LasR-dependent RhlR QS, in some isolates, it was not influenced by MexT activity, demonstrating that the clinical isolates had a similar architecture but may depend on a modulator other than MexT.

Our study identifies the mechanism of a key regulator of QS in *P. aeruginosa*. We originally hypothesized that QS regulation in PAO1 was mediated either by the efflux pump MexEF-OprN or the chaperone PqsE. However, our data suggest that it is the combination of both induction of *mexEF-oprN* expression and repression of *pqsE* expression, which drives the LasR-dependent RhlR QS observed in our strain. Several other studies have identified the QS signals C4-HSL, 3OC12-HSL, and HHQ as potential or established substrates of the efflux pump MexEF-OprN, supporting our observation that MexEF-OprN modulates QS (27, 34). Separate studies have shown that a suite of RhlR-regulated genes requires PqsE (36, 37). Our work shows a synergistic role between the two in regulating QS architecture. Furthermore, our data reveal that RhlR function modulation by MexEF-OprN is also influenced by a PqsE-independent pathway. We are exploring potential candidates in a PqsE-independent pathway by examining the MexT regulon we identified in this study.

Our laboratory and others have characterized QS extensively in strain PAO1 (2, 3, 25, 41), but we wanted to further explore the diversity of QS architecture in other genetic backgrounds. We previously identified LasR-independent QS in clinical isolates derived from chronic infections of people with cystic fibrosis (15). However, these isolates harbored *lasR* null mutations at the time of isolation, and it was unknown if they had developed a different architecture from PAO1 due to other genomic differences. We showed that clinical isolates can also have LasR-dependent QS like PAO1. Surprisingly, we observed varying degrees of dependence on LasR, ranging from complete dependence on LasR for RhlR activity in E194 and partial dependence on LasR for RhlR activity in E195. It will be of interest to explore the differences between the strains that result in differing degrees of dependence on LasR, including understanding how the level of PqsE activity contributes to phenotypes (42). Although there was dependence on LasR for RhlR activity in all clinical isolates, we discovered that this was not influenced by MexT. This result illustrates that while PAO1 and clinical isolates share similar QS hierarchies, the modulators of that architecture seem to be different. Identifying these additional modulators in other isolates might illuminate the determinants of alternate QS architectures.

Our study of MexT-mediated regulation of QS affords us some insight into the formation of QS hierarchies. Some bacteria contain only a single QS circuit, whereas others have many (2, 43, 44). Our study begins to explore why bacteria might have multiple QS circuits through understanding how MexT functions to modulate QS. We also identified unique QS architectures in the EPIC isolates, indicating that different regulators may be required for particular environments.

Additionally, this work offers insight into the plasticity of the interactions between QS circuits and how key regulators like MexT can alter QS architecture based on selective pressures and promote architectures that might be advantageous in specific environments. By studying and identifying variants in regulators like MexT, we gain insight into how cooperative behaviors may function within and between individuals. It is easy to imagine that regulatory mutants can change QS and cellular behaviors to allow for specialization within a community. For example, a *mexT* mutant may have a fitness advantage when RhlR activity is required early, whereas the wild-type defers the cost associated with RhlR QS, which might be advantageous when nutrients or other resources are limited.

Altogether, our study defined the regulation of a key modulator of *P. aeruginosa* growth and pathogenesis. By understanding how MexT modulates QS architecture, we begin to understand how bacterial sociality is controlled. These results have implications and applications that expand beyond bacterial pathogenesis and clinical settings. For example, how was MexT selected to regulate QS architecture in PAO1, but not the clinical isolates? What selective factors favored genetic changes that resulted in LasR-dependent QS with different architecture regulators? How do these regulators influence QS in the high percentage of *P. aeruginosa* isolates (from any environment) that are LasR-defective? Do the environment and other microbial interactions affect which architectures evolved (and which are beneficial in any given environment)? Our work pries the door open on understanding, where QS differs by interaction and environment, and what the modulators of these QS architectures are.

## MATERIALS AND METHODS

### Bacteria and growth conditions

Strains and plasmids used in this study are listed in Tables S5 and S6. *P. aeruginosa* was grown in lysogeny broth (LB) buffered with 50 mM 3-(*N*-morpholino) propanesulfonic acid, pH 7.0 (LB-MOPS). *E. coli* was grown in LB. Cultures were grown in 18 mm test tubes at a volume of 3 mL in a shaking incubator (250 RPM) at 37°C. For individual colony growth, we used LB supplemented with 1.5% agar. Where required, broth cultures of *E. coli* and *P. aeruginosa* were supplemented with gentamicin at a concentration of 10 µg/mL (Gm10). *E. coli* colonies were grown on LB supplemented with 1.5% agar and gentamicin at 10 µg/mL. *P. aeruginosa* colonies were grown on LB supplemented with 1.5% agar and gentamicin at 100 µg/mL (Gm100) for transformations or 10 µg per mL for maintaining cultures.

### Construction of *P. aeruginosa* mutants

In all experiments with the laboratory strain PAO1, we used strain *P. aeruginosa* PAO1-UW (45). Clinical *P. aeruginosa* isolates were obtained from the EPIC Observational Study and are from children with cystic fibrosis 5–12 years of age (39). In-frame deletions of *lasR*, *pqsE*, *mexT*, and *mexEF* were generated using two-step allelic exchange as previously described (46). Briefly, constructs for gene deletions were created by using a pEXG2 vector backbone and Gibson assembly to 1,000 bp of DNA flanking each side of the gene of interest to facilitate homologous recombination. *E. coli* S17-1 was transformed with each construct and used to deliver knockout plasmids to *P. aeruginosa* via conjugation. Merodiploids were selected by plating on *Pseudomonas* Isolate agar containing Gm100, and deletion mutants were then selected on LB agar containing 10%–15% sucrose and no sodium chloride. All deletion mutants were confirmed by PCR and sequencing of genomic DNA.

Overexpression constructs were created using a pUC18T mini Tn7T integrating plasmid with an arabinose-inducible promoter and Gibson assembly to clone in either *pqsE* or *mexEF-oprN* CDS downstream of the promoter (47). The overexpression construct was electrotransformed into *P. aeruginosa* as described elsewhere and selected using LB supplemented with 1.5% agar and gentamicin at 100 µg/mL (Gm100) (48). Proper integration at the attTn7 attachment site was confirmed by PCR and sequencing of genomic DNA. Gentamicin-susceptible mutants were made using Flp-mediated excision of the gentamicin resistance marker (49).

Reporter plasmids pP*_lasI_-gfp*, pP*_rhlA_-gfp*, pP*_pqsA_-gfp,* and pP*_mexE_-gfp* were made in pBBR1MCS-5 and contain about 200–500 bp upstream of each gene fused to *gfp*. Plasmids were electrotransformed into *P. aeruginosa* as described above.

### Assays for LasR, RhlR, and PqsR activity

To measure LasR activity, we inoculated individual duplicate colonies of strains with the transcriptional reporter P*_lasI_-gfp* into LB-MOPS supplemented with Gm10 and incubated at 37 ˚C with shaking for 18 h. Cultures were back diluted to an OD_600_ of 0.01 and grown until OD_600_ of 0.1. Exponential phase cultures were used to inoculate a 48-well plate to an OD_600_ of 0.01 with a 300 µL final volume. Plates were incubated at 37 ˚C with shaking for 18 h in a Biotek Synergy H1 microplate reader. GFP fluorescence (excitation 485 nm, emission 535 nm) and OD_600_ were measured every 20 min. The plasmids pP*_rhlA_-gfp* and pP*_pqsA_-gfp* were similarly introduced into each strain to measure RhlR and PqsR activity, respectively. Where appropriate, 20 µM final PQS (in methanol) was added before plates were incubated in the microplate reader. Similarly, to induce *pqsE* and *mexEF-oprN* expression, LB-MOPS supplemented with Gm10 was inoculated with a final concentration of 0.1% arabinose before plates were incubated in the microplate reader. All experiments were performed in triplicate.

### Assay for MexT activity

Strains were transformed with the pP*_mexE_-gfp* reporter plasmid (24). Single colonies were used to inoculate LB-MOPS supplemented with Gm10 and grown for 20 h. Cultures were back diluted to an OD_600_ of 0.05 in LB-MOPS supplemented with Gm10. To determine whether MexT activity can be activated, 8 mM final diamide [1,1′-azobis(N,N-dimethylformamide), TCI America] was added to cultures. Cultures were incubated for 18 h at 37˚C with shaking. Endpoint GFP fluorescence (excitation 485 nm, emission 535 nm), and OD_600_ were measured using a Biotek Synergy H1 microplate reader. For all endpoint measurements, the background fluorescence of LB was subtracted from the final fluorescence value of the reporter culture.

### RNA isolation and RNA-seq

Wild-type PAO1 and PAO1∆*mexT* cultures were started from single colonies in LB-MOPS in biological triplicate. Cultures were incubated for 18 h at 37˚C with shaking. Cultures were back diluted to an OD_600_ of 0.1 until cultures doubled, and then, the cultures were diluted back to an OD_600_ of 0.05. At an OD_600_ of 1.0 and 2.0, a total of an OD_600_ of 2.0 was pelleted at 4,000 RPM for 5 min. The supernatant was discarded, and the pellets were resuspended in 1 mL QIAzol containing lysis beads. Samples were lysed by bead-beating at maximum RPM for 1 min, with chilling for 5 min. Bead-beating was repeated twice. Chloroform was added, shaken vigorously, and centrifuged for 15 min at 12,000 × *g* at 4°C; 450 µL of the upper phase was combined with 675 µL of 100% ethanol. Samples RNA was extracted using an RNeasy kit with one on-column DNase (cat. No. 79254, Qiagen) treatment, and RNA was eluted using RNase-free water.

rRNA depletion, library generation, and > 20 million 150 bp paired-end Illumina HiSeq reads were generated for each sample commercially (Genewiz; Azenta). Trimmomatic with default settings (https://github.com/timflutre/trimmomatic) was used to trim adapters prior to alignment against the PAO1 reference genome (accession NC_002516) using StrandNGS version 3.3.1 (Strand Life Sciences, Bangalore, India). DESeq2 (31) was used for differential expression analysis, using the Benjamini-Hochberg adjustment for multiple comparisons and a false-discovery rate α = 0.05. Samples were grouped according to each strain and growth phase for the DESeq2 differential expression analyses. MexT regulons were determined by comparing the wild-type PAO1 with PAO1∆*mexT* for a given growth phase and imposing a 2-fold minimal fold change threshold. RhlR regulon genes were filtered out based on a compiled RhlR core regulon (15, 16, 32). The RNA-seq data are deposited in the Gene Expression Omnibus under accession no. GSE291609.

### qRT-PCR analysis

RNA was extracted as described above; 200 ng total cDNA was generated by reverse transcription using the qScript cDNA synthesis kit. Quantitative real-time PCR was performed using 2.5 ng total cDNA and the PowerTrack SYBR Green Mix. Primers targeting *mexE* were used to monitor MexT activity. Primers amplifying *rplU* were used as a housekeeping gene. Data analysis was determined using relative gene expression levels using the 2^-∆∆Ct^ method.

### Measurement of C4-HSL concentrations

Individual colonies of wild-type PAO1 or PAO1∆*mexEF* were individually inoculated into 3 mL LB-MOPS in triplicate. Cultures were grown at 37°C in a shaking incubator for 18 h. Cultures were back diluted to an OD_600_ of 0.1 and grown until OD_600_ of 0.2, and then back diluted to OD_600_ of 0.1. Cultures were grown at 37°C with shaking. At OD_600_ of 0.3, 0.8, and 2.0, cultures were removed to extract C4-HSL. To extract total C4-HSL, 3 mL of culture was transferred to an 18 mm glass test tube and mixed with 3 mL acidified ethyl acetate. Samples were vortexed on high for 30 s and rested for 10 min to allow for phase separation. The clear top layer was extracted into a 10 mL glass collection tube. The extraction was repeated and added to the same 10 mL glass collection tube. To extract intracellular C4-HSL, we centrifuged 10 mL of culture at 5,000 × *g* for 5 min, washed bacterial pellets three times with PBS, resuspended pellets in 2 mL LB-MOPS, and then added 2 mL acidified ethyl acetate. Samples were vortexed on high for 30 s and rested for 10 min to allow for phase separation. The clear top layer was extracted into a 10 mL glass collection tube. The extraction was repeated and added to the same 10 mL glass collection tube. For each sample, acidified ethyl acetate was evaporated using an N_2_ evaporator until no liquid remained. Dried fractions were resuspended in ethyl acetate to create a 10× stock.

To measure C4-HSL, we used a bioassay strain *E. coli* DH5α/pEDP61.5 containing *tacp rhlR* and a *rhlAB-lacZ* reporter (50). The bioassay strain was grown in 5 mL LB supplemented with 100 µg/mL carbenicillin for 18 h at 37°C with shaking. Cultures were back diluted to an OD_600_ of 0.05 and grown until an OD_600_ of 0.1–0.2. Cultures were induced with 1 mM final IPTG and grown to an OD_600_ of 0.5. C4-HSL standards and the above extracted samples were deposited into 1.5 mL microcentrifuge tubes and dried with N_2_. When cultures reached an OD_600_ of 0.5, 0.5 mL of culture was added to the prepared signal-containing tubes. Cultures were grown for 3 h at 37°C with shaking. Cultures were lysed with the addition of 50 µL chloroform, vortexed, and 10 µL of the top phase of each sample was transferred to a 96-well white microtiter Optiplate. Luminescence was measured using the Galacton-Plus chemiluminescent kit.

### Measurement of HHQ and PQS concentrations

For quantifications of HHQ and PQS, whole cultures were spiked with 5,6,7,8-tetradeutero-4-hydroxy-2-heptylquinoline (HHQ-d4) as an internal standard and extracted with ethyl acetate (51). Samples were injected using an HPLC Waters 2795 (Mississauga, ON, Canada) on a Kinetex C18 column (Phenomenex) with an acetonitrile-water gradient containing 1% acetic acid. The detector is a tandem quadrupole mass spectrometer (Quattro Premier XE; Waters) equipped with a Z-spray interface using electrospray ionization in positive mode (ESI+). Nitrogen was used as a nebulizing and drying gas at flow rates of 15 and 100  mL min^−1^. The following transitions were monitored in multiple reactions monitoring (MRM) mode: HHQ 244 → 159, HHQ-d_4_ 248 → 163, and PQS 260 → 175. The pressure of the collision gas (argon) was set at 2  ×  10^−3^ mTorr and the collision energy at 30 V.

### Measurement of pyocyanin production

Pyocyanin assays were performed with *P. aeruginosa* grown in LB-MOPS for 24 h at 37°C with shaking. Pyocyanin was extracted from 4 mL culture supernatant with 4 mL chloroform and was then extracted from the chloroform phase with an equal volume of 0.2 M hydrochloric acid-water. The absorbance was measured at 520 nm, and this value was converted to milligrams per milliliter of pyocyanin by multiplying the optical density at 520 nm (OD_520_) by 17.072 (52).

### Measurement of rhamnolipid production

To measure rhamnolipid production, we inoculated individual triplicate colonies of each strain into LB and incubated at 37°C with shaking for 18–20 h. The overnight cultures were standardized to 2.5 OD in 300 µL, and 20 µL was spotted onto methylene blue-containing rhamnolipid detection plates (0.6% [wt/vol] Na_2_HPO_4_, 0.3% [wt/vol] KH_2_PO_4_, 0.05% [wt/vol] NaCl, 1.5% [wt/vol] Noble agar, 0.1 mM CaCl_2_, 2 mM MgSO_4_, 0.2% glucose, 0.05% glutamate, 0.0005% methylene blue, 0.02% cetyltrimethylammonium bromide, and 0.05% casamino acids). Plates were incubated at 37˚C for 24 h and then transferred to a dark room temperature location for 48 h until a halo appeared. We measured the colony and halo area (mm^2^) and calculated the ratio of the halo formation to colony growth.

### Worm virulence assay

Wild-type Bristol N2 *Caenorhabditis elegans* strain VC2010 was maintained as detailed in the “Maintenance of *C. elegans*” chapter of wormbook.org. Briefly, *C. elegans* was maintained on nematode growth medium (NGM) agar (2.5 g/L Bacto-Peptone, 3 g/L NaCl, 17 g/L Bacto-Agar, 1 mM MgSO_4_, 25 mM KH_2_PO_4_, pH 6, 1 mM CaCl_2_, and 5 mg/L cholesterol) supplemented with *E. coli* OP50 as food (NGM-OP50). Worms were washed and suspended in M9W buffer (22 mM KH_2_PO_4_, 42 mM Na_2_HPO_4_, 86 mM NaCl, and 1 mM MgSO_4_). Virulence assays were conducted on phosphate-depleted slow kill agar (3.5 g/L Bacto-Peptone, 3 g/L NaCl, 17 g/L Bacto-Agar, 1 mM MgSO_4_, 1 mM CaCl_2_, and 5 mg/L cholesterol). The virulence assay was based on protocols described previously (53–55). Worms were passaged on fresh NGM-OP50 every 2–3 days prior to synchronization and maintained at room temperature. To generate synchronized early L4 worms, populations containing gravid adults were lysed with a freshly prepared alkaline hypochlorite solution (1.5 mL 10–15% NaOCl, 1.2 mL 3 M NaOH, and 4.8 mL H_2_O) to harvest eggs as described elsewhere (53). Egg suspensions were washed three times with M9W with centrifugation at 1,500 × *g* for 30 s between washes. Harvested eggs were suspended in 5 mL M9W and allowed to hatch for 24 h with gentle rotation at 18–20°C. Synchronized L1-arrested worms suspended in M9W were transferred to NGM-OP50 using low-bind tips and incubated at 18°C–20°C for 40–48 h until the early L4 growth stage. The early L4 growth stage of the worms was confirmed by observation with a dissecting microscope. On the day of L1 worm plating, 10 µL of overnight bacterial cultures were plated onto 35 mM plates with phosphate-depleted slow kill agar, and the plates were then incubated for 24 h at 37°C, after which they were incubated at 25°C overnight. For each bacterial strain, four replicate plates were included for each experiment. Synchronized early L4 worms were collected from NGM-OP50 agar plates by washing twice with M9W using centrifugation at 200 × *g* and then resuspended in 2 mL M9W. The worm suspensions were shaken slowly on a rocker at room temperature for 3–6 h. Prior to the addition of worms, 40 µL of 10 mg/mL 5-fluoro-2′-deoxyuridine (FUdR; Millipore Sigma) was added in small drops along the edge of the bacterial plates to prevent hatching of eggs during the assay. Ten to 20 early L4 worms were added to each bacterial plate. Plates were incubated at 25°C, and live worms were scored daily for 8 days.

## SUPPLEMENTAL MATERIAL

Fig. S1 mbio.02941-25-s0001.pdf Expression of *mexT* at the *att* site in PAO1Δ*mexT* restores regulation of RhlR.

Fig. S2 mbio.02941-25-s0002.pdf MexT is quiescent in clinical isolates and can be activated by diamide supplementation.

Tables S5 and S6 mbio.02941-25-s0003.docx Strains and plasmids.

Supplemental Tables mbio.02941-25-s0004.xlsx Tables S1 to S4.

ASM does not own the copyrights to Supplemental Material that may be linked to, or accessed through, an article. The authors have granted ASM a non-exclusive, world-wide license to publish the Supplemental Material files. Please contact the corresponding author directly for reuse.
