# Supplementary material for: Modulation of the Pseudomonas aeruginosa quorum sensing cascade by MexT-regulated factors
Source: mBio. 2025 Oct 23;16(11):e02941-25. doi: 10.1128/mbio.02941-25 (PMC12607905; doi:10.1128/mbio.02941-25)
Supplement: Fig. S1 — Expression of mexT at the att site in PAO1ΔmexT restores regulation of RhlR. [file mbio.02941-25-s0001.pdf]

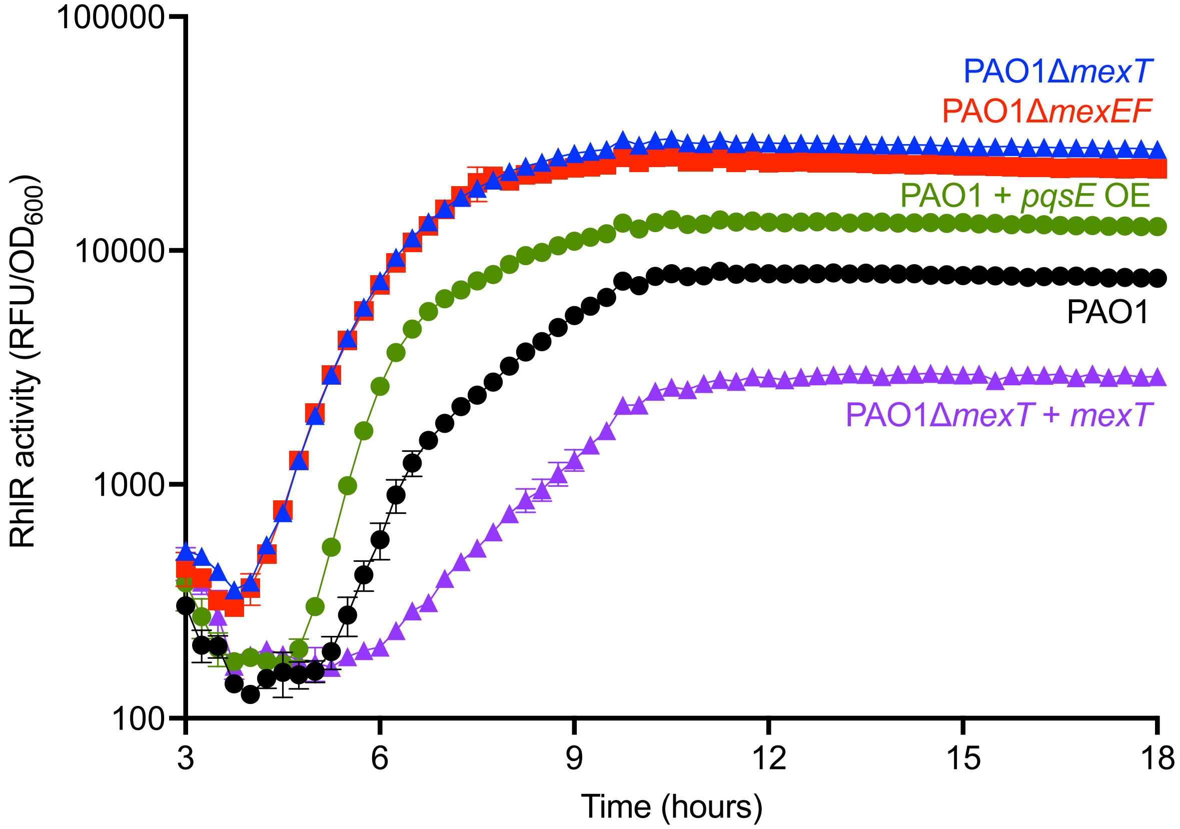

Supplemental Figure 1. Expressing *mexT* at the *att* site in PAO1Δ*mexT* restores regulation of RhlR. RhlR activity for PAO1, PAO1Δ*mexT*, PAO1Δ*mexEF*, and PAO1Δ*mexT* + *mexT* were determined using a RhlR activity reporter plasmid. *P* values were calculated using a two-way ANOVA with Geisser-Greenhouse correction where all strains were compared to wild-type PAO1. All unannotated comparisons met a $P < 0.05$.
