# Supplementary material for: Modulation of the Pseudomonas aeruginosa quorum sensing cascade by MexT-regulated factors
Source: mBio. 2025 Oct 23;16(11):e02941-25. doi: 10.1128/mbio.02941-25 (PMC12607905; doi:10.1128/mbio.02941-25)
Supplement: Fig. S2 — MexT is quiescent in clinical isolates and can be activated by diamide supplementation. [file mbio.02941-25-s0002.pdf]

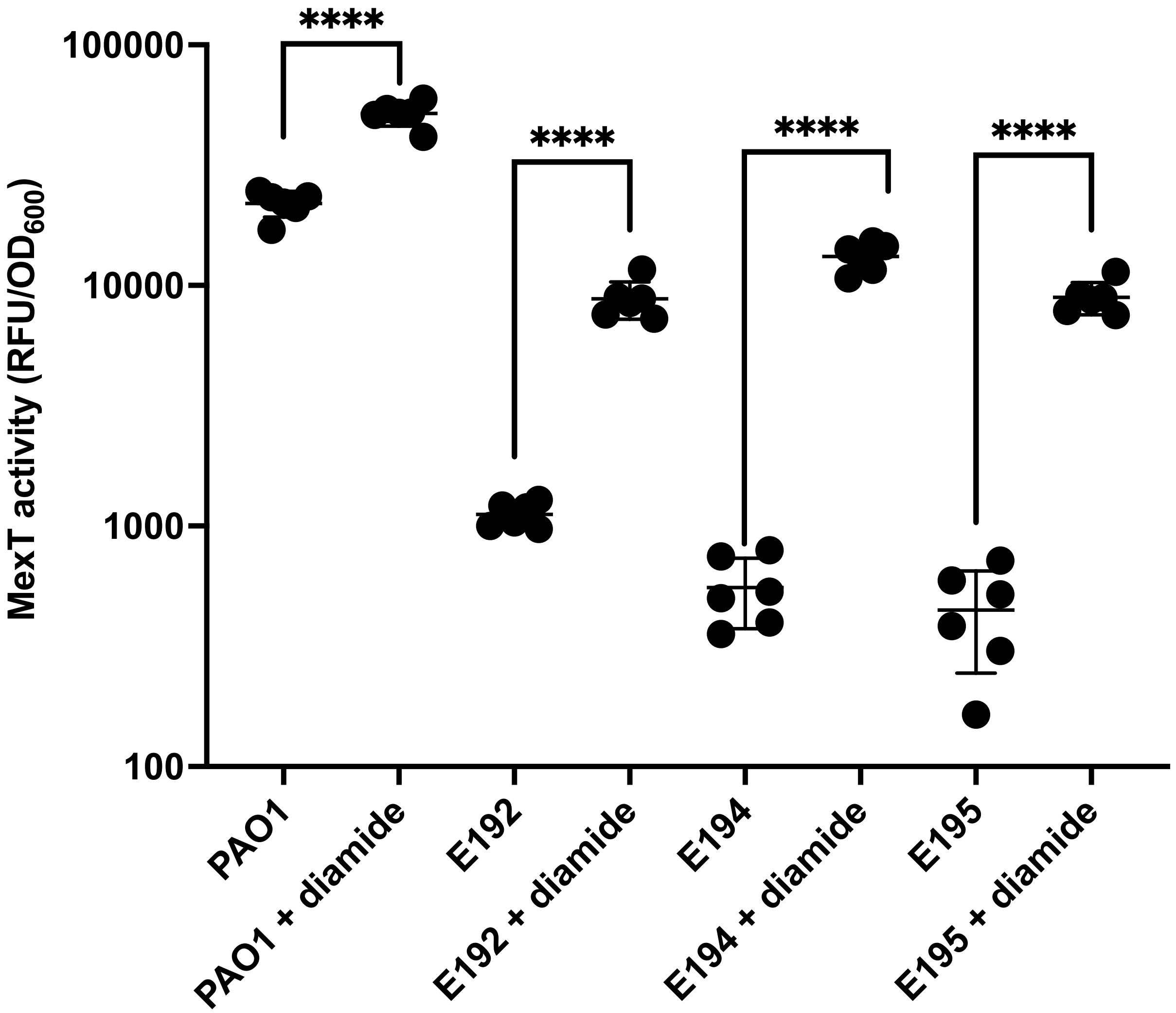

Supplemental Figure 2. MexT is quiescent in clinical isolates and can be activated by diamide supplementation. MexT activity for PAO1 and clinical isolates were determined using a MexT activity reporter plasmid. *P* values were calculated using an unpaired two-tailed t test with Welch's correction. **** denotes a *P* < 0.0001.
