# Supplementary material for: Modulation of the Pseudomonas aeruginosa quorum sensing cascade by MexT-regulated factors
Source: mBio. 2025 Oct 23;16(11):e02941-25. doi: 10.1128/mbio.02941-25 (PMC12607905; doi:10.1128/mbio.02941-25)
Supplement: Tables S5 and S6 — Strains and plasmids. [file mbio.02941-25-s0003.docx]

**Supplemental Tables**

**Supplemental Table 5. Bacterial strains used in this study.**

| Bacterial strain | Description | Source |
| --- | --- | --- |
| *P. aeruginosa* |  |  |
| PAO1 | Wild-type strain | [1] |
| PAO1∆*lasR* | PAO1 with unmarked, in-frame *lasR* deletion | [2] |
| PAO1∆*lasR ∆mexT* | PAO1 with unmarked, in-frame *lasR* and *mexT* deletion | This work |
| PAO1∆*mexT ∆pqsE* | PAO1 with unmarked, in-frame *mexT* and *pqsE* deletion | [3] |
| PAO1*∆mexT* | PAO1 with unmarked, in-frame *mexT* deletion | [3] |
| PAO1*∆mexEF* | PAO1 with unmarked, in-frame *mexEF* deletion | This work |
| PAO1 attn7::P_araBAD_-*pqsE* | Made by introducing pUC18-miniTn7T-*araBAD* *pqsE* to PAO1 | This work |
| PAO1 attn7::P_araBAD_-*mexEF-oprN* | Made by introducing pUC18-miniTn7T-*araBAD* *mexEF-oprN* to PAO1 | This work |
| E192 | Cystic fibrosis lung clinical *P. aeruginosa*  isolate | [4] |
| E192 ∆*lasR* | E192 with unmarked, in-frame *lasR* deletion | This work |
| E192 ∆*mexT* | E192 with unmarked, in-frame *mexT* deletion | This work |
| E192 ∆*lasR ∆mexT* | E192 with unmarked, in-frame *lasR* and *mexT* deletion | This work |
| E194 | Cystic fibrosis lung clinical *P. aeruginosa*  isolate | [4] |
| E194 ∆*lasR* | E194 with unmarked, in-frame *lasR* deletion | This work |
| E194 ∆*mexT* | E194 with unmarked, in-frame *mexT* deletion | This work |
| E194 ∆*lasR ∆mexT* | E194 with unmarked, in-frame *lasR* and *mexT* deletion | This work |
| E195 | Cystic fibrosis lung clinical *P. aeruginosa*  isolate | [4] |
| E195 ∆*lasR* | E195 with unmarked, in-frame *lasR* deletion | This work |
| E195 ∆*mexT* | E195 with unmarked, in-frame *mexT* deletion | This work |
| E195 ∆*lasR ∆mexT* | E195 with unmarked, in-frame *lasR* and *mexT* deletion | This work |
|  |  |  |
| *E. coli* |  |  |
| NEB5α | *fhuA2Δ(argF-lacZ)U169 phoA glnV44 Φ80Δ(lacZ)M15 gyrA96 recA1 relA1* | NEB |
| S17-1 | *recA pro hsdR RP4-2Tc::Mu-Km::Tn7* | [5] |

**Supplemental Table 6. Plasmids used in this study.**

| Plasmids | Description | Source |
| --- | --- | --- |
| pEXG2 | Allelic exchange vector with pBR origin, *sacB*, Gm^R*^ | [6] |
| pEXG2-PAO1-*mexT-*KO | pEXG2 containing sequences for PAO1 *mexT* in-frame deletion | [7] |
| pEXG2-PAO1-*mexEF-*KO | pEXG2 containing sequences for PAO1 *mexEF* in-frame deletion | This work |
| pEXG2-E192-*lasR-*KO | pEXG2 containing sequences for E192 *lasR* in-frame deletion | This work |
| pEXG2-E192-*mexT-*KO | pEXG2 containing sequences for E192 *mexT* in-frame deletion | This work |
| pEXG2-E194-*lasR-*KO | pEXG2 containing sequences for E194 *lasR* in-frame deletion | This work |
| pEXG2-E194-*mexT-*KO | pEXG2 containing sequences for E194 *mexT* in-frame deletion | This work |
| pEXG2-E195-*lasR-*KO | pEXG2 containing sequences for E195 *lasR* in-frame deletion | This work |
| pEXG2-E195-*mexT-*KO | pEXG2 containing sequences for E195 *mexT* in-frame deletion | This work |
| pBBR1MCS-5 | Broad-host-range expression plasmid, Gm^R^ | [8] |
| pP*_lasI_*-gfp | pBBR1MCS-5 with *lasI* promoter fused to *gfp*, Gm^R^; encodes -282 to +223 relative to the start of *lasI* and includes the complete *rsaL* binding site | [3] |
| pP*_rhlA_*-gfp | pBBR1MCS-5 with *rhlA* promoter fused to *gfp*, Gm^R^; encodes -500 to +31 relative to the start of *rhlA* | [3] |
| pP*_pqsA_*-gfp | pBBR1MCS-5 with *pqsA* promoter fused to *gfp*, Gm^R^; encodes -429 to +3 relative to the start of *pqsA* | [9] |
| pP*_mexE_*-gfp | pBBR1MCS-5 with *mexE* promoter fused to *gfp*, Gm^R^; encodes -500 to +31 relative to the start *mexE* | [3] |
| pUC18-mini-tn7T-*araBAD* | Suicide delivery vector with insertion at attTn7 site, Gm^R^ | This study |
| pTNS2 | T7 transposase expression vector; R6K *ori*, *ori* T, Amp^R**^ | [10] |
| pFLP2 | Site-specific excision vector; *sacB*, *ori* T, Cb^R***^ | [11] |
| pUC18-mini-tn7T-*araBAD pqsE* | pUC18-mini-Tn7T-*araBAD* with P*_araBAD_*-driven *pqsE* | This study |
| pUC18-mini-tn7T-*araBAD mexEF-oprN* | pUC18-mini-Tn7T-*araBAD* with P*_araBAD_*-driven *mexEF-oprN* | This study |

* Gm^R^, resistant to gentamicin

** Amp^R^, resistant to ampicillin

*** Cb^R^, resistant to carbenicillin

Supplemental references

1. Stover, C.K., et al., *Complete genome sequence of Pseudomonas aeruginosa PAO1, an opportunistic pathogen.* Nature, 2000. **406**(6799): p. 959-64.

2. Wang, M., et al., *Quorum sensing and policing of Pseudomonas aeruginosa social cheaters.* Proc Natl Acad Sci U S A, 2015. **112**(7): p. 2187-91.

3. Kostylev, M., et al., *Relationship of the transcription factor MexT to quorum sensing and virulence in Pseudomonas aeruginosa.* J Bacteriol, 2023. **205**(12): p. e0022623.

4. Feltner, J.B., et al., *LasR Variant Cystic Fibrosis Isolates Reveal an Adaptable Quorum-Sensing Hierarchy in Pseudomonas aeruginosa.* mBio, 2016. **7**(5).

5. Simon, R., U. Priefer, and A. Puhler, *A Broad Host Range Mobilization System for Invivo Genetic-Engineering - Transposon Mutagenesis in Gram-Negative Bacteria.* Bio-Technology, 1983. **1**(9): p. 784-791.

6. Rietsch, A., et al., *ExsE, a secreted regulator of type III secretion genes in Pseudomonas aeruginosa.* Proc Natl Acad Sci U S A, 2005. **102**(22): p. 8006-11.

7. Kostylev, M., et al., *Evolution of the Pseudomonas aeruginosa quorum-sensing hierarchy.* Proc Natl Acad Sci U S A, 2019. **116**(14): p. 7027-7032.

8. Kovach, M.E., et al., *Four new derivatives of the broad-host-range cloning vector pBBR1MCS, carrying different antibiotic-resistance cassettes.* Gene, 1995. **166**(1): p. 175-6.

9. Smalley, N.E., et al., *Evolution of the Quorum Sensing Regulon in Cooperating Populations of Pseudomonas aeruginosa.* mBio, 2022. **13**(1): p. e0016122.

10. Choi, K.H., et al., *A Tn7-based broad-range bacterial cloning and expression system.* Nat Methods, 2005. **2**(6): p. 443-8.

11. Hoang, T.T., et al., *A broad-host-range Flp-FRT recombination system for site-specific excision of chromosomally-located DNA sequences: application for isolation of unmarked Pseudomonas aeruginosa mutants.* Gene, 1998. **212**(1): p. 77-86.
